# Escalation of Ethanol Drinking in Mice Is Associated With Neurochemical Changes in the Dorsal Striatum

**DOI:** 10.1111/adb.70101

**Published:** 2025-11-25

**Authors:** Eric Baetscher, Timothy L. Carlson, Connor Hilts, Jade L. Thomas, Patrick N. Reardon, Verginia C. Cuzon Carlson, Christopher D. Kroenke

**Affiliations:** ^1^ Department of Biomedical Engineering Oregon Health and Science University Portland Oregon USA; ^2^ Advanced Imaging Research Center Oregon Health and Science University Portland Oregon USA; ^3^ Division of Neuroscience Oregon National Primate Research Center Beaverton Oregon USA; ^4^ Oregon State University NMR Facility Oregon State University Corvallis Oregon USA

**Keywords:** alcohol, ethanol, glutamate, glutamine, magnetic resonance spectroscopy, mice, striatum

## Abstract

Prior studies indicate that a bias towards excitation in the excitatory/inhibitory balance occurs in the dorsal striatum as a result of chronic heavy alcohol use. However, investigations of the dorsal striatum by noninvasive means, such as magnetic resonance spectroscopy (MRS) in vivo, have been lacking. In this study, 49 mice (27 female) on a C57BL/6J genetic background were subject to a chronic intermittent ethanol and forced swim stress (CIE‐FSS) procedure. For each mouse, MRS data were acquired at two timepoints from a single voxel covering the dorsal striatum bilaterally. The baseline MRS timepoint occurred in an ethanol naïve state. Ethanol drinking for all mice consisted of 1‐h/day access to one bottle of 15% ethanol, for 5 days/week. After a 6‐week ethanol drinking acclimation period, mice were assigned to four experimental groups: air and no stress, air and stress, EtOH_vapour_ and no stress, and EtOH_vapour_ and stress. Mice then underwent four cycles of CIE‐FSS, each cycle consisted of 1 week of vapour (air or ethanol) followed by 1 week of either 10 min of FSS swim stress or no swim stress, and 1‐h access to 15% ethanol. A follow‐up MRS timepoint occurred at the conclusion of the 14‐week CIE‐FSS protocol, after at least 43 h of abstinence from ethanol. Whole‐cohort ethanol drinking increased from a mean ± SEM of 1.51 ± 0.11 g/kg at Week 1, to a mean of 3.15 ± 0.13 g/kg at Week 14. Robust increases in several neurochemicals were observed between baseline and follow up, with the largest increases found for glutamate, glutamine, taurine, and creatine. Two sets of analysis on MRS data were performed: (1) assessing effects of experimental group (CIE, FSS and/or sex) on striatal neurochemistry and (2) investigating correlations between longitudinal changes in ethanol drinking and changes in neurochemical concentrations. Striatal neurochemical concentrations exhibited CIE‐FSS group effects (1), notably EtOH_vapour_ exposure significantly reduced lactate concentrations at follow up. The change in glutamate between baseline and follow up was significantly lower in EtOH_vapour_ exposed mice. Changes in the glutamate/glutamine ratio were markedly reduced in the EtOH_vapour_ condition. In our correlational analysis (2) of changes in drinking versus changes in neurochemical concentrations from baseline to follow up, we found that female mice have strong positive linear correlations between the change in ethanol drinking and the changes in concentration of glutamate, glutamine, taurine, myo‐inositol and creatine. In male mice, inverse correlations were found between changes in drinking and changes in glutamate, taurine and creatine concentrations. Additionally, the change in cerebral ventricle volume was found to correlate with the change in ethanol drinking, but ventricular volume change was not significantly affected by CIE or FSS. Together, the observed effects of CIE on MRS outcomes in dorsal striatum and the correlations between neurochemical changes and drinking behaviour substantiate the importance of the dorsal striatum in escalating ethanol consumption and are promising evidence for the utility of MRS to detect behaviourally relevant changes in heavy ethanol drinking phenotypes.

## Graphical abstract

The dorsal striatum of mice exposed to ethanol and stress was examined by magnetic resonance spectroscopy. Sex‐dependent correlations were observed between the change in ethanol consumed and the change in several neurochemicals, including glutamate.

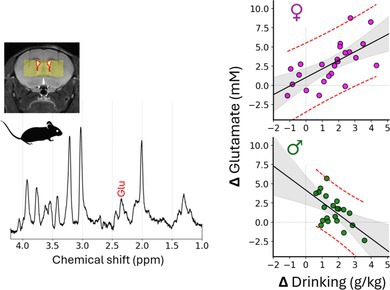

## 1 Introduction

The dorsal striatum is a critical brain region in the neural circuitry of addiction [1, 2, 3]. Electrophysiological studies report shifts towards increased excitatory (glutamatergic) compared with inhibitory (GABAergic) neural activity associated with chronic ethanol (EtOH) exposure in this region [4, 5, 6]. The ability to noninvasively detect neurophysiological changes within the dorsal striatum during the emergence of heavy ethanol drinking behaviour would improve understanding of how this neural circuitry contributes to the biological basis of addiction. Chronic ethanol exposure and stress are risk factors for heavy ethanol consumption [7, 8, 9, 10]. An experimental procedure that enables the separate contributions of stress and ethanol exposure to ethanol drinking separately, and in combination, involves chronic intermittent ethanol exposure combined with forced swim stress (CIE‐FSS). The CIE‐FSS experiment has reliably demonstrated escalation of ethanol drinking in mice dependent on CIE and FSS exposure, along with interactions between the two factors. Interestingly, robust increases in drinking have resulted from ethanol vapour and stress exposed male mice [11, 12, 13], but more variable effects on drinking have been observed in females [14, 15]. Sex differences in the effects of ethanol and behaviours associated with ethanol drinking are widely reported [16, 17, 18], including differences in MRS outcomes [19, 20]. Therefore, this study was designed to examine sex differences in general; however, it was largely exploratory with respect to possibly divergent sex‐dependent MRS outcomes.

Magnetic resonance spectroscopy (MRS) is a noninvasive analytical method that can be used to quantify neurochemical concentrations in specific brain regions. Notably, glutamate (the primary excitatory neurotransmitter) and GABA (the principal inhibitory neurotransmitter) are among the neurochemicals measurable by MRS in the brain [21, 22]. Interest in obtaining a neuronal excitatory‐to‐inhibitory ratio with MRS has previously been operationalized by calculating the ratio of glutamate over GABA [23, 24, 25, 26], and in this study, we likewise investigate the Glu/GABA ratio in the dorsal striatum. Preclinical MRS studies in rats report neurochemical changes in response to stress [27, 28], and specific changes in the basal ganglia following ethanol exposure have been described [29, 30, 31, 32, 33]. Prior findings have also indicated that neurochemicals measured by MRS are associated with motivated behaviours, in both humans and preclinical models [34, 35, 36, 37, 38]. Prior MRS studies addressing the neurobiology of alcohol exposure have largely focused on cortical regions, particularly the anterior cingulate cortex [29, 31, 32, 39, 40, 41, 42], and the few MRS studies of the striatum have targeted the nucleus accumbens [32, 41, 43].

The objective for this study was to utilize in vivo MRS to assess whether neurochemical changes in the dorsal striatum are related to ethanol and/or stress exposure in male and female mice, thereby testing the hypothesis that neurochemical measurements by MRS are correlated with factors related to drinking behaviour. Glutamate and GABA concentration changes throughout the experimental procedures were specifically assessed within the dorsal striatum, to determine whether MRS changes recapitulate increased excitatory neural tone previously reported in studies using electrophysiology [4, 5, 6]. In addition, the rich neurochemical information present in longitudinal MRS data and the variation in drinking behaviour exhibited by mice that undergo the CIE‐FSS procedure provide the opportunity to broadly assess neurometabolic consequences in the dorsal striatum related to ethanol and stress exposure, and ultimately to ethanol drinking patterns.

## 2 Methods

### 2.1 Animal Procedures

All animal procedures were performed with approval from the Oregon Health and Science University (OHSU) Institutional Animal Care and Use Committee and supervised by veterinarians from the OHSU Department of Comparative Medicine. Forty‐nine (27 females) pvalb‐Cre mice on a C57BL/6J background (Stock #: 17320, Jackson Laboratory, Bar Harbor, ME, USA) arrived at our facility at 8 weeks of age. Mice were intracranially injected with 300 nL of adeno‐associated virus solution (44 361‐AAV2, Addgene, Watertown, MA, USA), with titres of ~10^12^ viral genomes per millilitre, targeting the dorsolateral striatum (stereotaxic coordinates: AP + 0.55 mm, ML ± 2.2 mm, DV −3.25 mm from bregma), to express the hM3Dq excitatory designer receptor under the Cre‐recombinase expressed in parvalbumin‐expressing interneurons. The results reported here are for conditions in which the hM3Dq receptors were inactive, with components of this study involving activated hM3Dq receptors to be reported elsewhere.

**FIGURE 1:**
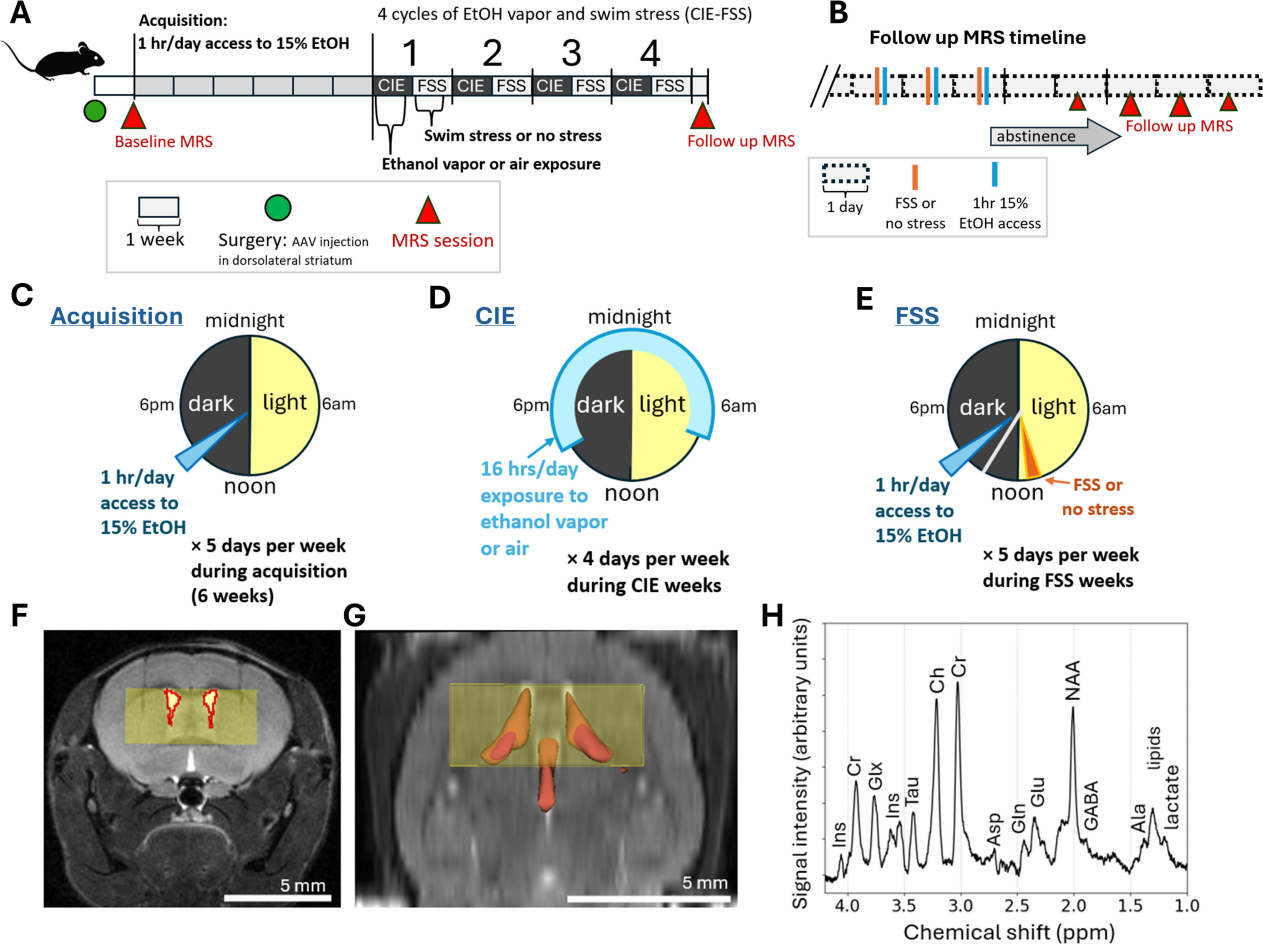
The timeline of the overall experiment (A) consisted of the following: surgical intracranial injection of an adeno‐associated viral vector designed to cause expression of the excitatory designer receptor hM3Dq in parvalbumin expressing interneurons in the dorsolateral striatum of the mice (green circle). Once each mouse recovered from surgery for a minimum of 3 days, a baseline MRS scan was performed. Subsequently, mice underwent 6 weeks of ethanol drinking acquisition, followed by experimental group assignment. Four cycles of chronic intermittent ethanol and forced swim stress (CIE‐FSS) were conducted over the following 8 weeks, and finally a follow up MRS scan was performed between 43 and 120 h after the last session of ethanol drinking; however, > 75% of the mice were scanned after between 60 and 100 h of abstinence (B). The follow‐up MRS timepoint for each mouse was immediately followed by euthanasia, including collection of brain tissue for high resolution NMR spectroscopy. During acquisition (C), mice were provided with one bottle of 15% w/v ethanol for one hour per day, beginning 3 h after the initiation of the dark phase of the modified light/dark cycle. At the conclusion of the ethanol consumption acquisition phase, four treatment groups were established and balanced by sex and ethanol consumption during acquisition. The four groups were: air + no stress, air + stress, EtOH vapour + no stress, EtOH vapour + stress. Four cycles of CIE‐FSS were then performed. During the first week of each CIE‐FSS cycle, mice were placed in vapour exposure chambers and exposed to either ethanol vapour or air during 4 days of the CIE week (D). During the second week of each CIE‐FSS cycle, mice in the stress condition were subjected to 10 min of swimming in 25°C water on 5 days/week (E). During these FSS weeks, all mice voluntarily consumed ethanol for 1 h/day, and this consumption was recorded to assess escalation of EtOH drinking behaviour and to investigate correlates of heavy drinking behaviour. (F) Coronal T_2_‐weighted RARE MR image with representative PRESS voxel position (yellow rectangle), placed to contain the dorsal striatum. Segmented ventricular overlap with the MRS voxel is indicated by red borders. (G) A representative MRS voxel displayed in the axial plane with a volume rendering of the segmented ventricles demonstrating degree of overlap with the MRS voxel. (H) Representative ^1^HMR spectrum acquired from the PRESS voxel indicated in the left panel. Ala, alanine; Asp, aspartate; Ch, total choline; Cr, creatine; GABA, gamma‐aminobutyric acid; Gln, glutamine; Glu, glutamate; Glx, glutamate + glutamine; Ins, myo‐inositol; NAA, N‐acetyl aspartate; Tau, taurine.

Approximately 1 week after surgery, mice received a baseline MRS session and then began the CIE‐FSS protocol (Figure 1A). All mice underwent a 6‐week ethanol drinking acquisition phase in which mice had 1 h/day (5 days/week) access to 15% ethanol (v/v), beginning 3 h into the dark cycle (Figure 1C). Mice were then assigned to one of four groups, balanced for sex and mean ethanol drinking during Week 6 of acquisition. Experimental groups were (1) air with no stress (−, −), (2) air with swim stress (−, +), (3) EtOH_vapour_ with no stress (+, −) and (4) EtOH_vapour_ with swim stress (+, +). Following acquisition, mice then underwent four cycles of CIE in which each cycle consisted of 1 week of ethanol/air vapour and 1 week of stress/no stress. The vapour week consisted of 16 h/day, 4 days/week exposure to ethanol vapour or air (Figure 1D). The ethanol vapour administration produced mean ± SEM blood ethanol concentrations (BECs) of 140.5 ± 10.5 mg/dL for 24 mice in the EtOH_vapour_ condition. Mice in the control group were placed in vapour chambers but were exposed only to room air. Prior to being placed in vapour chambers, EtOH_vapour_ mice received an injection of 1‐mmol/kg pyrazole and 1.6‐g/kg ethanol (I.P.) while control air mice received an intraperitoneal (I.P.) injection of 1 mmol/kg of pyrazole in saline. For stress/no stress weeks, mice in the stress groups underwent FSS in which mice were placed in a 2‐L plastic beaker filled ~50% with room temperature water for 10 min, starting 1 h prior to the dark cycle, 5 days/week. Following FSS, mice were removed from the water, dried with a paper towel and placed in their home cage atop a heating pad. Control mice remained in their home cages. Mice were then given access to 1 bottle of 15% ethanol (v/v) for 1 h, 3 h into the dark cycle (Figure 1E). Twice a week, 1 h prior to the start of the 1‐h ethanol drinking period, mice received an I.P. injection of either the DREADDs actuator deschloroclozapine (100 μg/kg) or saline. The order of injections was counterbalanced across mice. All ethanol drinking data for FSS weeks presented here are from the 3 days without I.P. injections. The 15% ethanol bottles were weighed at the start and end of the 1‐h ethanol drinking period. The difference was recorded as the volume consumed and was further corrected for handling and evaporation by subtraction of the mean volume loss from two control bottles placed in empty cages on each drinking day.

### 2.2 Magnetic Resonance Experiments

A baseline MRS session was performed on each mouse prior to the initiation of the 6‐week ethanol drinking phase of the experiment. A follow‐up MRS study was conducted on each mouse after the fourth (and final) cycle of CIE‐FSS, 43 to 120 h after the most recent ethanol drinking session (Figure 1B). MRS data were acquired using a 12‐Tesla MRI instrument (*Bruker Biospin*, Billerica, MA, USA). A circularly polarized mouse head radiofrequency coil (*m2m*, Cleveland, OH, USA) was utilized for all MR measurements. Mice were anaesthetised with 1%–2% isoflurane to maintain a respiratory rate of ~100 breaths per minute, and body temperature was maintained at 37°C with a forced‐air heater. A T_2_‐weighted RARE MRI sequence (acquisition time [TA] = 3m44s, repetition time [TR] = 3500, echo time [TE] = 8.6 ms, number of averages [NA] = 2, slices = 20, resolution = 0.098 × 0.098 × 0.5 mm) was acquired to facilitate placement of a point‐resolved spectroscopy (PRESS) voxel over the dorsal striatum. The PRESS voxel contained a volume of 37.5 μL (6 left/right × 2.5 dorsal/ventral × 2.5‐mm rostral/caudal) and was positioned ventral to the medial corpus callosum and caudal to the anterior commissure (Figure 1F,G). MRS voxels were segmented into ventricle and grey matter classes using a semi‐automated intensity‐based approach.

Each PRESS series was acquired with settings of TE = 25 ms, TR = 2.5 s, 8192 complex points, spectral bandwidth = 5000 Hz, NA = 128, TA = 5 m 20 s. An unsuppressed water spectrum was acquired to obtain water‐normalized concentration values for the metabolites of interest. Subsequent water‐suppressed PRESS spectra were then acquired with variable power and optimized relaxation delays (VAPOR) water suppression [44]. To minimize the effects of chemical‐shift displacement of the MRS voxel in the spectral region containing signals from the metabolites of interest, the transmitter carrier frequency of the MR system was adjusted to be set 1225 Hz upfield of the water resonance (2.25 PPM). A representative 25‐ms TE PRESS spectrum is shown in Figure 1H.

### 2.3 Brain Tissue Extraction NMR Spectroscopy

To obtain information about the tissue concentrations of neurochemicals, at the conclusion of the follow‐up MRS session, mice were euthanized with a large dose of isoflurane and perfused with ice‐cold artificial cerebrospinal fluid (CSF). Brains were removed and the dorsal striatum from the right hemisphere was dissected, weighed and immediately transferred to a cryogenic sample tube and submerged in liquid nitrogen. These brain tissue samples were then stored at −80°C until aqueous metabolite extraction was performed. The biochemical analysis was focused on the right hemispheres because the left hemispheres underwent fixation procedures for immunohistochemical analyses.

A solution of sodium trimethylsilylpropanesulfonate (DSS) (*Chenomx Inc*., Edmonton, AB, Canada) was added to each tissue sample at 2‐μmol DSS per gram of tissue (wet weight) as a concentration and chemical shift reference. After the addition of the DSS solution, tissue was immediately homogenized with a Potter‐Elvehjem homogenizer. A chloroform–methanol–water extraction was then performed following previously described methods [45]. The aqueous fraction was transferred to a new microcentrifuge tube and dried at room temperature using a vacuum centrifuge dryer. The dried samples were resuspended in 99.9% deuterium oxide. Nuclear magnetic resonance (NMR) spectroscopy data were acquired on a Bruker Avance III 800‐MHz spectrometer with a cryogenic probe at Oregon State University using a presaturation‐NOESY pulse sequence with Chenomx recommended parameters (spectral bandwidth = 12PPM, recycle delay = 1 s, NA = 512). These solution‐state spectra were quantified using the *Chenomx* software suite.

### 2.4 Data Analysis

MRS data were analysed using LCModel version 6.3‐1R [46]. A basis set with 18 neurochemicals was generated using the FID‐A MATLAB library [47]. In addition, nine lipid and macromolecular signals were part of the LCModel basis set. Only neurochemical estimates with a Cramér–Rao lower bound less than or equal to 20% and with high‐quality fitting assessed by visual inspection were included in subsequent analyses. Eleven neurochemicals from the basis set were quantified with acceptable reliability for statistical analysis across both timepoints: Alanine (Ala), Aspartate (Asp), gamma‐aminobutyric acid (GABA), glutamine (Gln), glutamate (Glu), myo‐inositol (Ins), lactate (Lac), taurine (Tau), total choline (Cho), N‐acetyl aspartate (NAA) and total creatine (Cr). We also performed statistical analysis on two neurochemical ratios of interest: Glu/GABA and Glu/Gln.

We used a semi‐automated intensity‐based approach to segment the lateral ventricles (excluding the temporal horns) and the third ventricle and further determined the region of the lateral ventricle volume within the MRS voxel in each mouse (Figure 1G). Neurochemical tissue concentrations were then corrected for the fraction of the MRS voxel containing ventricular CSF (mM_corrected_ = mM_raw_ / [1 − *ventricle fraction of MRS voxel*]). To achieve absolute neurochemical concentration estimates in vivo, we used NAA concentrations from high‐resolution NMR spectroscopy of the homogenized tissue extracts, correcting our water‐normalized in vivo concentration estimates for bias introduced by water referencing. NAA was chosen as the normalization reference due to reported high stability in aqueous tissue extraction preparations [48]. This correction factor was determined to be 0.4378 and was multiplied by each in vivo water‐referenced neurochemical estimate.

Within‐mouse differences from baseline to follow up for each neurochemical were calculated and used to assess longitudinal concentration changes. These differences were examined at the whole‐cohort level with one‐sample *t*‐tests with correction for multiple comparisons. Statistical testing of the relationship between the MRS‐measured neurochemical changes and ethanol consumption changes utilized ordinary least squares linear regression. Group comparisons for each of the quantified neurochemicals and fit T_2_ values were conducted by three‐way ANOVA. These analyses were implemented in Python using the *statsmodels* library [49]. Statistical significance was defined as *p* < 0.05. All data are presented as mean ± SEM.

## 3 Results

### 3.1 Ethanol Drinking Patterns

**FIGURE 2:**
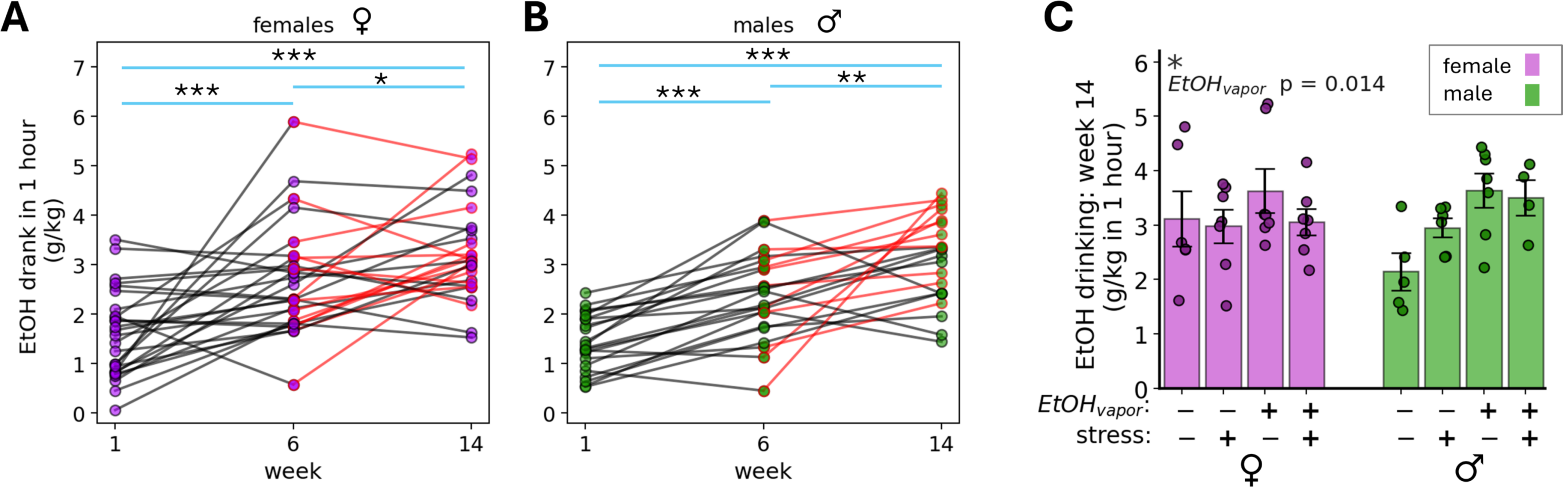
Both female (A) and male (B) mice exhibited escalating ethanol drinking across the 14 weeks of the experiment, visualized at three sentinel weeks in the experimental timeline: the first week of EtOH drinking prior to group assignment, the final week of the EtOH acquisition phase of the experiment (Week 6) and the final week of the CIE‐FSS procedure (Week 14). For the change in drinking from Week 1 to Week 14, within‐subjects *t*‐tests show an increase for the whole cohort: *t*(48) = −9.53, *p* = 1.2E‐12; for female mice: *t*(26) = −5.63, *p* = 6.35E‐6; and for male mice: *t*(21) = −10.45, *p* = 8.9E‐10. For the change in drinking from Week 1 to Week 6, the whole cohort significantly increased drinking: *t*(48) = −6.17, *p* = 1.38E‐7; as did female mice: *t*(26) = −3.94, *p* = 0.0006; and male mice: *t*(21) = −6.41, *p* = 2.38E‐6. For the change in drinking from Week 6 to Week 14, the whole cohort exhibited a significant increase: *t*(48) = −3.95, *p* = 0.0003; as did female mice: *t*(26) = −2.54, *p* = 0.018; and male mice: *t*(21) = −3.03, *p* = 0.006. Female mice generally exhibited more intra‐individual variability in drinking behaviour, and male mice showed a stronger increase in drinking over the final 8 weeks of the experiment compared with female mice. In the final cycle of CIE‐FSS (Cycle 4). Red lines between Week 6 and Week 14 indicate mice in the EtOH_vapour_ condition. (C) Mice in the EtOH vapour condition drank more alcohol compared with air‐exposed controls (*p* = 0.014). This treatment effect was mainly driven by male mice. Error bars depict standard error of the mean (SEM). *p < 0.05, **p < 0.01, ***p < 0.001.

Both the 6‐week drinking acquisition and 8‐week CIE‐FSS phases of the experiment (total of 14 weeks) occurred between the baseline and follow‐up MRS measurements (Figure 1A). Therefore, drinking during both phases was analysed for associations with neurochemical concentration changes. Drinking levels during the 1‐h/day access periods escalated between Weeks 1 and 6, as well as between Weeks 6 and 14, in males and females (Figure 2A,B). During the first week of ethanol drinking, average 1‐h ethanol consumption for female mice was 1.58 ± 0.17 g/kg and for male mice was 1.42 ± 0.12 g/kg. By Week 6, consumption had increased to 2.67 ± 0.21 g/kg for females and 2.34 ± 0.18 g/kg for males, and Week 14 drinking levels were 3.20 ± 0.18 g/kg for females and 3.09 ± 0.19 g/kg for males. Escalation was significant for both sexes by paired‐sample *t*‐tests (males: Weeks 1 to 6: *p* < 0.001; Weeks 6 to 14: *p* = 0.006; females: Weeks 1 to 6: *p* < 0.001; Weeks 6 to 14: *p* = 0.018). Females displayed larger increases, as well as greater inter‐individual drinking variability throughout the experiment (Figure S1). In the final CIE‐FSS cycle, ethanol vapour‐exposed mice consumed significantly more alcohol than air‐exposed controls by three‐way ANOVA (main effect of CIE, *p* = 0.014), with this treatment effect primarily driven by drinking patterns in male mice. No significant effects were found for forced swim stress (FSS) or group interactions (Figure 2C). Average drinking values for males, females and all mice during the final CIE‐FSS cycle are presented in Table 1.

**TABLE 1:** One‐hour ethanol drinking by experimental group (CIE‐FSS Cycle 4).

Group	Overall mean g/kg (SEM) [count]	Male mean g/kg (SEM) [count]	Female mean g/kg (SEM) [count]
Air + no stress	2.68 (0.34) [11]	2.15 (0.35) [5]	3.12 (0.51) [6]
Air + stress	2.97 (0.18) [13]	2.95 (0.17) [6]	2.98 (0.31) [7]
EtOH_vapour_ + no stress	3.63 (0.25) [14]	3.64 (0.31) [7]	3.63 (0.41) [7]
EtOH_vapour_ + Stress	3.22 (0.20) [11]	3.50 (0.33) [4]	3.06 (0.24) [7]

*Note:* Three‐way ANOVA detected a main effect of EtOH_vapour_ on drinking (*p* = 0.014).

### 3.2 Neurochemical Changes From Baseline

**FIGURE 3:**
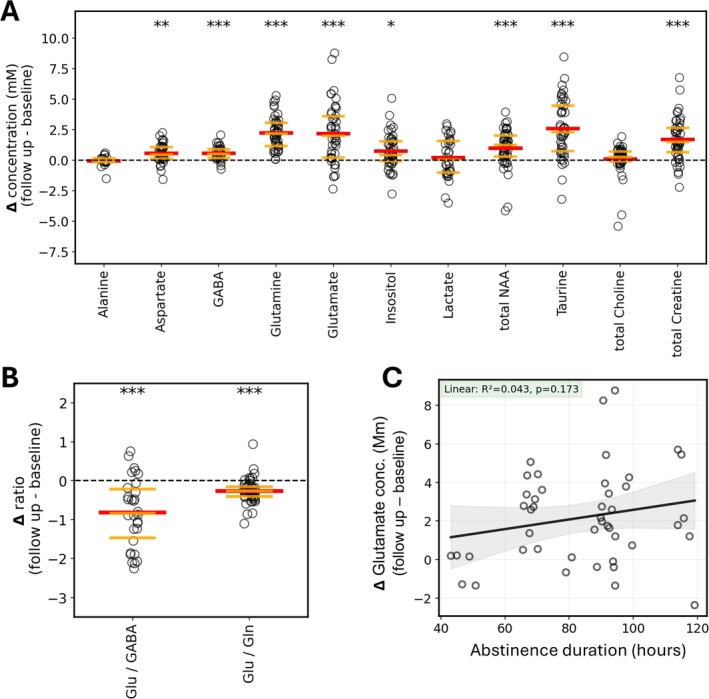
Differences between baseline and follow‐up MRS sessions for each mouse and 11 neurochemical concentration estimates that were reliably quantified in this cohort (A). One sample *t*‐tests for difference from zero indicate that 8 of the 11 neurochemicals significantly increased in concentration from baseline to follow up. Red bars indicate mean change; orange bars indicate boundaries between quartiles. Bonferroni correction applied to *p* values. Changes in two neurochemical ratios (B) were also investigated, with the ratio of glutamate over GABA for each mouse on average decreasing from baseline to follow up. While glutamate typically increased more than GABA in terms of mM, the mean Glu/GABA ratio at follow up was 4.02, therefore an increase of GABA of 1/4 that of glutamate would lead to an unchanged ratio from baseline to follow up. Similarly, the ratio of glutamate over glutamine showed a significant decrease on a whole‐cohort basis across the experiment. The effects of MRS abstinence timing on neurochemical concentration changes were also explored by linear regression (C). For within‐mouse changes in glutamate, a linear model explained 4.3% of the variance in Δglutamate (*R* ^2^ = 0.043, *p* = 0.173). Detailed analysis of abstinence effects is presented in Figure S2. *p < 0.05, **p < 0.01, ***p < 0.001.

To assess the cohort‐wide effects of the experimental protocol, we calculated the difference in neurochemical concentrations between baseline and follow‐up scans for each mouse and performed one‐sample *t*‐tests for difference from zero, with Bonferroni corrections for multiple comparisons (Table 2, Figure 3A,B). Eleven molecules and two ratios (Glu/GABA and Glu/Gln) were evaluated. Eight of the chemicals exhibited increases in concentration in the dorsal striatum between baseline and follow‐up: aspartate (mean change = 0.56 mM, *p* = 0.0002), GABA (mean change = 0.55 mM, *p* = 2.29E‐5), glutamine (mean change = 2.05 mM, *p* = 3.47E‐10), glutamate (mean change = 1.87 mM, *p* = 4.06E‐7), inositol (mean change = 0.72 mM, *p* = 0.001), NAA (mean change = 0.98 mM, *p* = 7.27E‐5), taurine (mean change = 2.34 mM, *p* = 1.43E‐8), total creatine (mean change = 1.57 mM, *p* = 3.87E‐8), glutamate/GABA ratios (mean change = −0.82, *p* = 2.03E‐5) and glutamate/glutamine ratios (mean change = −0.27, *p* = 2.04E‐7). Alanine, lactate and total choline did not significantly change from baseline. The reduction in the glutamate/GABA ratio despite a larger mean increase in glutamate (1.87 mM for Glu vs. 0.55 mM for GABA) is attributable to the original ratio of glutamate/GABA being 4.78 ± 0.73 at baseline. For each unit change in GABA, glutamate would need to increase by an additional factor of approximately 4.8 for the ratio to remain unchanged. In other words, with GABA increasing by, on average, 0.55 mM, glutamate would need to change by 2.64 mM for the ratio to remain unchanged. Consequently, the observed mean increase in glutamate of 1.87 mM leads to a reduction in the Glu/GABA ratio.

**TABLE 2:** Neurochemical concentrations and changes.

Neurochemical	Baseline mean (SEM) concentration mM	Follow up mean (SEM) concentration mM	Mean (SEM) within‐mouse change from baseline mM	*p* for difference from zero of concentration changes
Alanine (Ala)	1.00 (0.11)	0.88 (0.03)	−0.07 (0.12)	0.56
Aspartate (Asp)	2.21 (0.15)	2.62 (0.10)	0.56 (0.14)	**0.00024**
GABA	1.53 (0.10)	2.02 (0.06)	0.55 (0.11)	**2.29E‐5**
Glutamine (Gln)	3.64 (0.25)	5.73 (0.21)	2.05 (0.26)	**3.47E‐10**
Glutamate (Glu)	5.76 (0.29)	7.93 (0.15)	1.87 (0.31)	**4.06E‐7**
Myo‐inositol	3.66 (0.16)	4.45 (0.10)	0.72 (0.21)	**0.001**
Lactate	2.43 (0.16)	2.72 (0.15)	0.21 (0.30)	0.49
NAA	3.36 (0.24)	4.44 (0.09)	0.98 (0.23)	**7.27E‐5**
Taurine	4.89 (0.32)	7.66 (0.19)	2.34 (0.33)	**1.43E‐8**
Total choline	1.60 (0.16)	1.67 (0.03)	0.07 (0.17)	0.68
Total creatine	4.02 (0.25)	5.60 (0.10)	1.57 (0.24)	**3.87E‐8**
Glu/GABA	4.78 (0.12)	4.02 (0.08)	−0.82 (0.16)	**2.03E‐5**
Glu/Gln	1.71 (0.05)	1.45 (0.04)	−0.27 (0.04)	**2.04E‐7**

*Note:* Bold and underlined numbers indicate *p* values that are significant at *α* = 0.05.

Previous in vivo MRS studies of rodents exposed to high levels of ethanol have reported high‐amplitude but transient changes in neurochemistry as a consequence of withdrawal from ethanol [31, 41]. These findings are consistent with microdialysis measurements of biochemical changes in the brain extracellular space [50, 51]. Although a minimum of 9 days elapsed between the final vapour exposure and MRS measurement, the mice in this study also received 1‐h access to 15% ethanol between 2 and 5 days preceding follow‐up MRS (Figure 1A,B). Therefore, although acute withdrawal effects had likely resolved by the follow‐up MRS timepoint, it was of interest to determine whether neurochemical concentrations were affected by the duration of abstinence prior to follow‐up MRS measurements. It was also of interest to determine whether the observed association between neurochemical changes and changes in drinking behaviour could arise from abstinence‐related effects.

To address the first question, changes in neurochemical concentrations for each mouse were analysed as a function of hours since the last 1‐h ethanol drinking session, as shown for glutamate in Figure 3C, and for other neurochemicals, Glu/GABA and Glu/Gln in Figure S2. Significant associations were not observed in any of these comparisons. Moreover, the slopes of nonsignificant linear trends calculated from the data are generally opposite to the expected abstinence‐related glutamate dynamics, with acute elevation during the first day of abstinence followed by reduction towards normal concentrations over the following days. Next, to address whether abstinence‐related concentration changes could lead to spurious observed relationships with drinking behaviour, simulations were performed that examined the possibility that an underlying effect of abstinence duration may be present; however, it was not detected in our experiment, due to insufficient statistical power. For molecules that exhibit correlations with drinking behaviour (glutamate, glutamine, taurine and total creatine), we performed permutations over the set of actual abstinence durations, then modified the measured concentrations by adding simulated abstinence effects to produce *R* ^2^ values of 0.01, 0.05, 0.10, 0.15 and 0.20, as shown in Figure S3. It was found that increasing the effect size for abstinence did not appreciably affect the measured association between change in neurochemical concentration and change in ethanol drinking. These two analyses indicate that the MRS effects observed in this study are not consequences of transient neurochemical changes due to withdrawal and that these associations likely reflect longer‐term ethanol‐related allostasis.

### 3.3 Effects of Experimental Group and Sex

**FIGURE 4:**
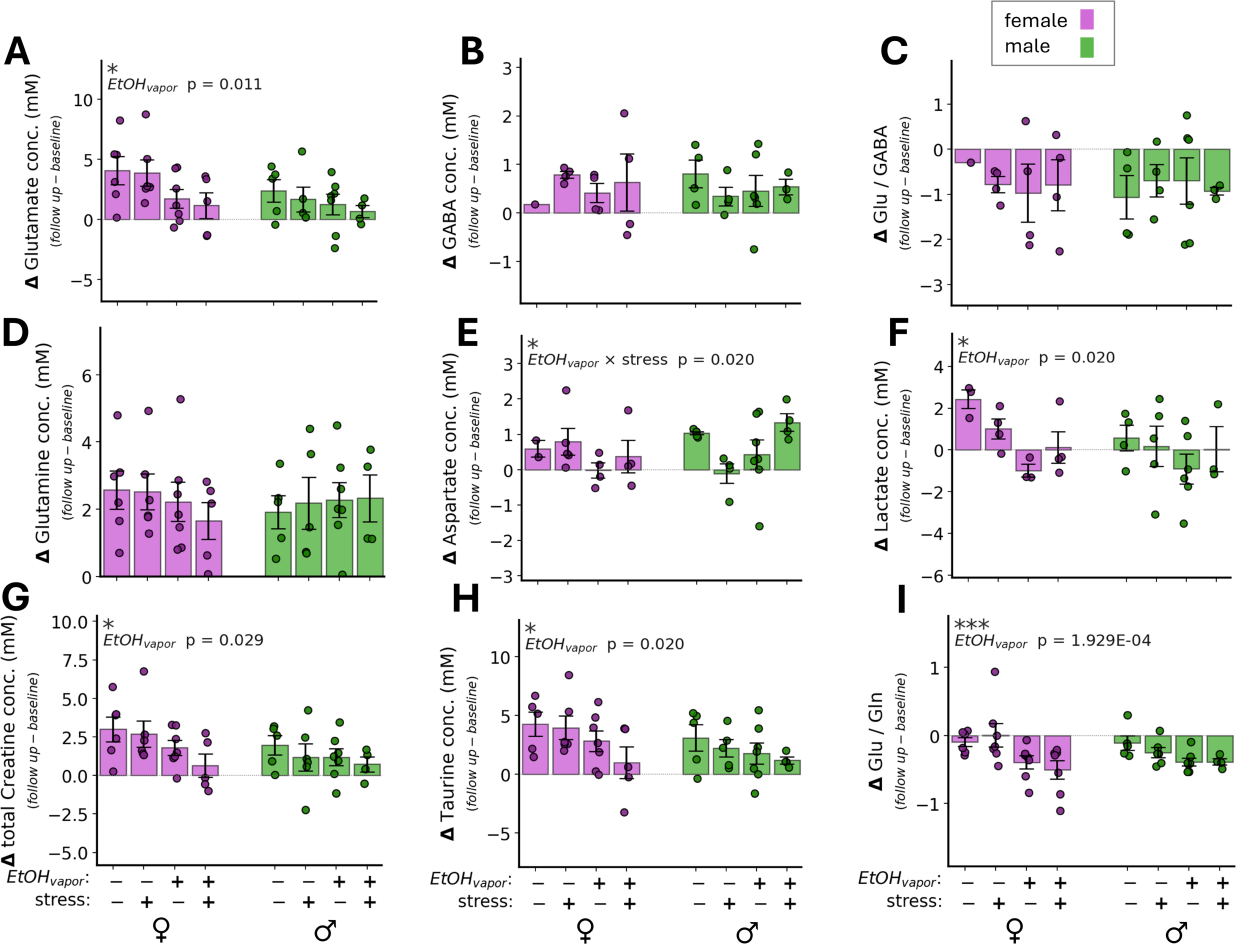
Within‐mouse neurochemical concentration changes from baseline to follow‐up by experimental group. The dashed line indicates zero change. Changes in glutamate (A) were lower in EtOH_vapour_ exposed mice compared with controls (*p* = 0.01). Changes for GABA (B), the glutamate to GABA ratio (C) and glutamine (D) did not show group differences. The change in aspartate (E) exhibited a significant CIE‐by‐FSS interaction (*p* = 0.02), similar to the effect observed on the follow‐up concentrations. Likewise, the change in lactate was significantly affected by EtOH_vapour_ (*p* = 0.02), with CIE‐exposed mice having on average lower lactate at follow‐up than at baseline and air‐exposed mice having higher lactate at follow‐up than at baseline. Two other quantified neurochemicals were significantly affected by CIE: Total creatine (G) (*p* = 0.03) and taurine (H) (p = 0.02) were lower in the EtOH_vapour_ condition. Finally, the change in the ratio of glutamate over glutamine exhibited a significant effect of EtOH_vapour_, (*p* = 1.93 × 10^−4^), with CIE‐exposed mice having a robust decrease in Glu/Gln compared with control mice. *p < 0.05, *** *p* < 0.001.

Within‐mouse neurochemical changes from baseline (follow up minus baseline) were calculated, and effects of sex, CIE and FSS were investigated by three‐way ANOVA. A main effect of CIE was found for changes in glutamate, inositol, lactate, taurine, total creatine and the ratio of glutamate/glutamine (Figure 4). The change in glutamate was significantly less positive (*F*[1,37] = 7.08, *p* = 0.011) in EtOH_vapour_ exposed mice compared with those exposed to air. A similar pattern was present for inositol, lactate, taurine and total creatine (Table 3, Figure 4). The strongest CIE effect was observed for the change in glutamate/glutamine ratio (*F*[1,41] = 16.78, *p* = 0.00019). The median change in glutamate/glutamine ratio was negative for all groups; however, the change was more negative in EtOH_vapour_ exposed mice. The pattern of group effects on the change in lactate was similar to the group effects observed on the lactate concentration at follow up only, providing additional support for the finding that lactate is decreased in EtOH_vapour_ exposed mice. The change in aspartate also showed a similar pattern as follow up aspartate concentrations alone (CIE by FSS interaction, *F*[1,27] = 6.05, *p* = 0.021). Lastly, a main effect of FSS was detected for the change in total choline concentration (*F*[1,41] = 4.31, *p* = 0.044).

**TABLE 3:** Results from ANOVA of group effects on changes in metabolite concentrations.

Neurochemical	CIE		FSS		Sex		CIE by FSS		CIE by sex		FSS by sex		CIE by FSS by sex	
	*F*‐statistic	*p*	*F*‐statistic	*p*	*F*‐statistic	*p*	*F*‐statistic	*p*	*F*‐statistic	*p*	*F*‐statistic	*p*	*F*‐statistic	*p*
Alanine	0.01	0.94	0.00	0.97	0.61	0.45	3.20	0.11	0.29	0.60	0.12	0.74	5.61	**0.04**
Aspartate	0.09	0.77	0.16	0.69	0.62	0.44	6.05	**0.02**	3.51	0.07	0.31	0.58	3.03	0.09
GABA	0.14	0.72	0.01	0.94	0.06	0.80	0.16	0.70	0.00	0.98	0.83	0.37	0.77	0.39
Glutamine	0.20	0.65	0.05	0.83	0.05	0.83	0.20	0.65	1.00	0.32	0.31	0.58	0.03	0.86
Glutamate	7.08	**0.01**	0.51	0.48	3.02	0.09	0.01	0.93	1.05	0.31	0.03	0.85	0.03	0.86
Inositol	4.73	**0.04**	2.02	0.16	0.88	0.35	0.05	0.81	1.79	0.19	0.00	0.96	1.49	0.23
Lactate	6.16	**0.02**	0.01	0.91	1.39	0.25	2.84	0.10	1.39	0.25	0.16	0.69	0.28	0.60
Taurine	5.90	**0.02**	1.74	0.20	2.04	0.16	0.23	0.63	0.46	0.50	0.06	0.81	0.42	0.81
Total choline	3.34	0.07	4.30	**0.04**	0.03	0.85	0.90	0.35	1.62	0.21	1.24	0.27	1.74	0.19
NAA	3.23	0.08	1.47	0.23	0.98	0.33	0.06	0.80	2.10	0.15	0.19	0.67	0.95	0.33
Total creatine	5.10	**0.03**	1.84	0.18	2.64	0.11	0.08	0.77	0.91	0.34	0.01	0.93	0.34	0.56
Glu/GABA	0.00	0.97	0.01	0.92	0.00	0.99	0.03	0.86	0.12	0.74	0.00	0.95	0.58	0.45
Glu/Gln	16.78	**0.00**	0.21	0.65	0.32	0.57	0.13	0.72	1.41	0.24	0.18	0.67	1.25	0.27

*Note:* Bold and underlined numbers indicate *p* values that are significant at *α* = 0.05.

The results from the follow‐up MRS session are presented in Table S1 and Figure S3. At follow‐up, lactate was found to exhibit a main effect of CIE treatment, with lower lactate concentrations observed in EtOH_vapour_‐exposed mice (*F*[1,35] = 4.77, *p* = 0.036) (Table S1, Figure S4). Aspartate was affected by the interaction of CIE and FSS (*F*[1,41] = 6.10, *p* = 0.018), with EtOH_vapour_ and stress‐exposed males having the highest mean aspartate concentrations of the eight groups. Glutamine at both baseline and follow‐up was higher in male mice than female mice (baseline: *F*[1,44] = 4.87, *p* = 0.039; follow‐up: *F*[1,41] = 7.27, *p* = 0.010). Finally, the glutamate/glutamine at follow‐up also displayed a main effect of sex (*F*[1,41] = 7.19, *p* = 0.010), largely driven by the sex differences in the glutamine factor in that ratio.

### 3.4 Correlations Between Neurochemical Changes and Ethanol Drinking

**FIGURE 5:**
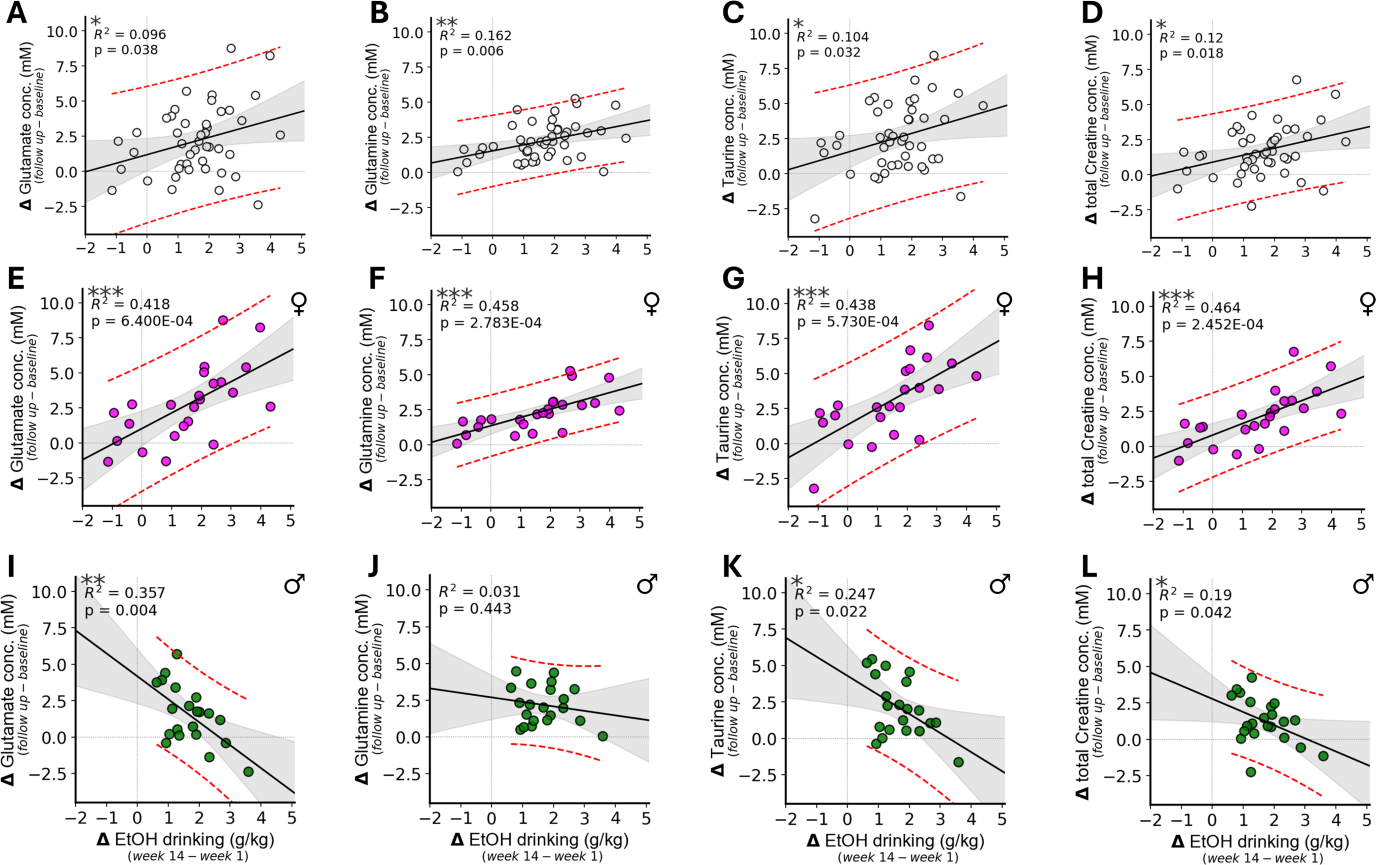
Correlations between changes in ethanol drinking behaviour and changes in select neurochemicals for whole cohort (top row, open makers), female mice (middle row, purple makers) and male mice (bottom row, green markers). Changes in glutamate concentrations (left column) were significantly and positively correlated with changes in drinking behaviour for the whole cohort and for female mice (A,E); however, male mice (I) exhibited an inverse correlation between drinking changes and glutamate changes. Changes in glutamine concentrations (B,F,J) were positively correlated with changes in drinking for the whole cohort and female mice but not for male mice. For changes in taurine (C,G,K) positive correlations for the whole cohort and female mice were observed, whereas male mice exhibited an inverse trend. For changes in total creatine (D,H,L), the whole cohort and female mice showed positive correlations while male mice demonstrated a weak inverse correlation. Black lines denote linear regression lines. Shaded regions indicate 95% confidence intervals of the regression lines; red dashed lines indicate the 95% confidence interval of the model predictions. *p < 0.05, **p < 0.01, ***p < 0.001.

In addition to the group‐based analyses presented above, potential associations between MRS findings and drinking were investigated on the individual animal level. Changes in ethanol consumption (from Week 1 to Week 14) of individual mice were compared with neurochemical changes from baseline to follow‐up. When analysed at the level of the whole group (Figure 5, top row), glutamate, glutamine, inositol, taurine and total creatine exhibited significant correlations between concentration change from baseline and ethanol drinking change from baseline (Glu: *R* ^2^ = 0.096, *p* = 0.038; Gln: *R* ^2^ = 0.16, *p* = 0.006; Ins: *R* ^2^ = 0.095, *p* = 0.036; Tau: *R* ^2^ = 0.10, *p* = 0.032; Cr: *R* ^2^ = 0.12, *p* = 0.018).

Interestingly, the relationship between changes in ethanol drinking and changes observed by MRS differs between males and females. For female mice (Figure 5, middle row), several neurochemical changes demonstrated sizeable correlations with ethanol drinking behaviour. Glutamate (*R* ^2^ = 0.42, *p* = 6.40E‐4), glutamine (*R* ^2^ = 0.46, *p* = 2.78E‐4), inositol (*R* ^2^ = 0.34, *p* = 0.002), taurine (*R* ^2^ = 0.44, *p* = 5.73E‐4) and total creatine (*R* ^2^ = 0.46, *p* = 2.45E‐4) were the neurochemicals exhibiting significant correlations with drinking behaviour. Notably, all these correlations for female mice were positive with respect to the change in the amount of ethanol voluntarily consumed (Week 14–Week 1 for each mouse). In contrast, there were fewer significant correlations in this analysis for male mice only, and the slope coefficients for male mice were negative for all neurochemicals (Figure 5, bottom row). Significant correlations with changes in ethanol drinking were observed for glutamate (*R* ^2^ = 0.357, *p* = 0.004), taurine (*R* ^2^ = 0.247, *p* = 0.022) and creatine (*R* ^2^ = 0.19, *p* = 0.042). Notably, all three of these correlations have negative slopes with respect to change in ethanol drinking, in contrast to the positive correlations for female mice.

### 3.5 Ventricular Volume Changes

**FIGURE 6:**
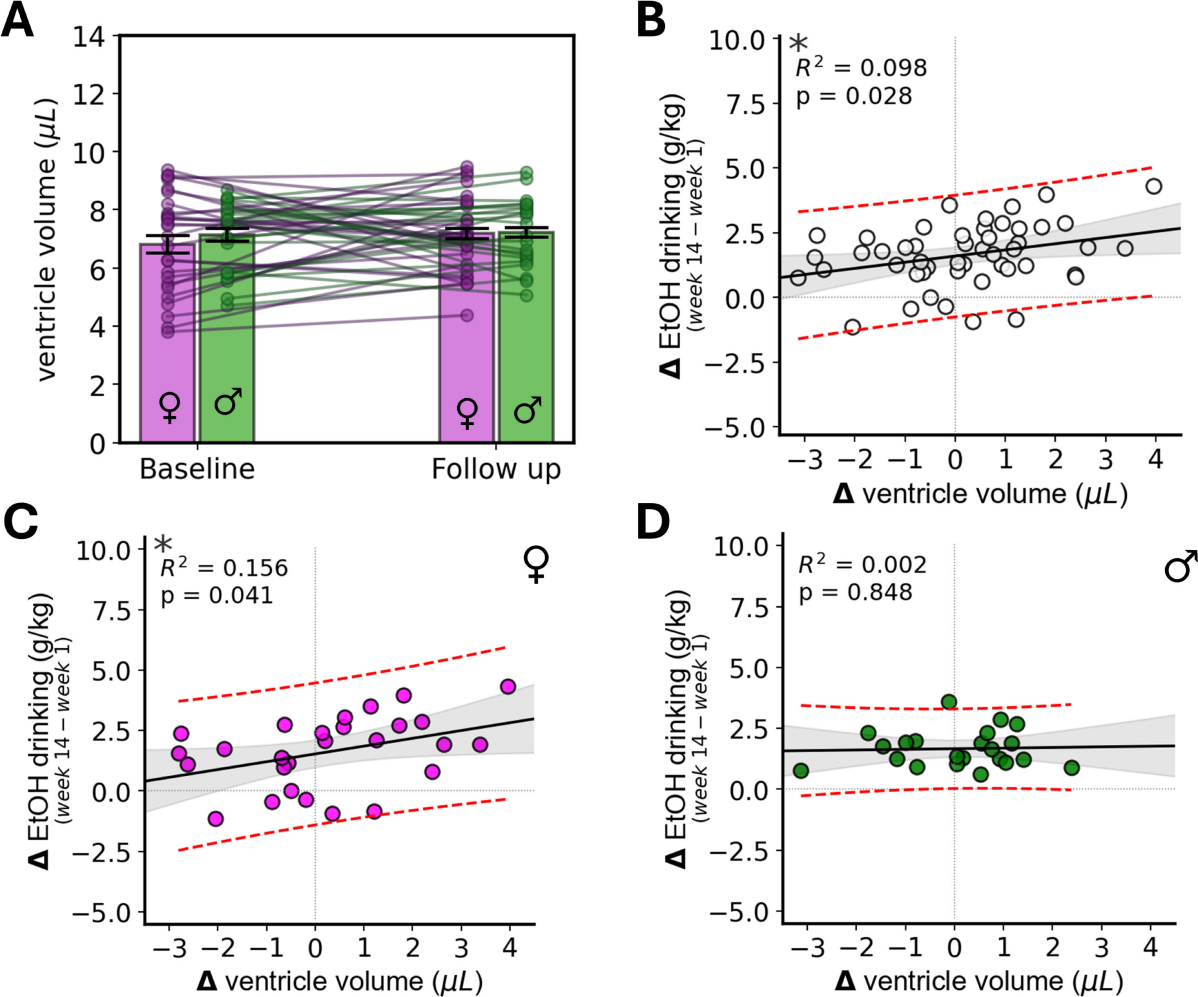
Analysis of ventricular volumes. No significant changes from baseline in ventricular volume were observed (A). The change in ventricle volume correlates with the change in drinking across the experiment (B,C) for the whole cohort (*R* ^2^ = 0.098, *p* = 0.028), and for female mice (*R* ^2^ = 0.156, *p* = 0.041), but not for male mice (*R* ^2^ = 0.002, *p* = 0.848) (D). Linear regression results are denoted by black lines. Shaded regions indicate 95% confidence intervals of regression lines; red dashed lines indicate 95% confidence intervals of the model predictions. *p < 0.05.

Studies by Zahr et al. have reported changes in ventricular volumes in rodent models of alcohol use [52, 53]. Moreover, the significant concentration differences we found between baseline and follow‐up for several neurochemicals motivated us to better understand brain water and CSF content in this cohort. As such, we assessed changes in ventricular volume between MRS timepoints as a function of sex, experimental group, and as a correlate with drinking behaviour. Analysis of the overall segmented ventricular volume revealed no significant changes from baseline to follow‐up for the whole cohort or when separated by sex (Figure 6A). No significant effects of experimental group on change in ventricular volume were found. However, changes in ventricle volume were positively correlated with changes in drinking from Week 1 to Week 14, with mice exhibiting greater increases in ventricle size having larger increases in ethanol drinking. The correlation between change in ventricular size and change in ethanol drinking was significant for the whole cohort (*R* ^2^ = 0.098, *p* = 0.028) (Figure 6B) and for females (*R* ^2^ = 0.16, *p* = 0.041) (Figure 6C), but not for males (*R* ^2^ = 0.002, *p* = 0.85) (Figure 6D).

## 4 Discussion

The objective of this study was to use in vivo MRS in mice that undergo chronic ethanol and/or stress exposure to assess how neural circuitry changes associated with heavy ethanol drinking relate to neurochemical changes in the dorsal striatum. The dorsal striatum is mostly composed of GABAergic neurons, with 90%–95% of the total neurons being medium spiny neurons and < 5% being GABAergic interneurons [54, 55]. Glutamatergic axonal input into the dorsal striatum arises mostly from the cortex and thalamus. Based on reports from electrophysiological experiments that the relative excitatory and inhibitory tone in the dorsal striatum is disrupted by heavy drinking [4, 56], a primary focus of this study was on the neurotransmitters glutamate and GABA. In this experiment, daily ethanol intake generally increased over the 14‐week procedures, but specific effects of CIE and FSS on drinking were modest. Nevertheless, the overall findings were that widespread changes in neurochemical concentrations occurred, including glutamate and GABA, and that these changes were closely associated with ethanol intake, but with qualitatively different associations in male compared with female mice.

### 4.1 Brain Changes Include, but Are Not Limited to, Alterations in Glutamate and GABA Concentrations

Prior MRS literature reports alterations of glutamate and GABA resulting from ethanol exposure in cortical and ventral striatal brain regions [39, 40, 41, 43]. Studies using other methodologies but focused on the dorsal striatum have indicated that dorsal striatal neural activity becomes increasingly excitatory as a result of sensorimotor cortical projection activity during chronic ethanol exposure [4, 5, 6, 57, 58]. Our MRS data complement these findings by directly monitoring both glutamate and GABA concentration changes that take place between a baseline measurement, prior to ethanol exposure, and a follow‐up measurement acquired at the conclusion of the experiment. Both neurotransmitters increased in concentration between baseline and follow‐up. Unexpectedly, the ratio of glutamate to GABA decreased overall.

There are fundamental differences between MRS and neurophysiological measures. Although electrophysiological techniques primarily detect synaptic activity, MRS is sensitive to multiple neurochemical pools, including intracellular neural and astrocytic compartments, in addition to neurotransmitters in the extracellular and synaptic spaces. This distinction may help explain apparent discrepancies between these methodologies and emphasizes that MRS is sensitive to concentration changes due to metabolic processes as well as changes in neurotransmitter activity. Furthermore, neurochemical changes were not limited to alterations in neurotransmitters. Total choline, total creatine, NAA and taurine also exhibited changes similar to those found for glutamate and GABA. The widespread effects of the experimental procedures on the concentration of multiple biochemical constituents measured by MRS suggest that changes in metabolic processes beyond excitatory and inhibitory synaptic signalling occur in the mouse striatum.

This broad pattern of alterations may reflect a more fundamental change in brain tissue water content, as MRS measurements are referenced to water signal amplitudes, and concentration is defined by solvent volume. To further assess brain water content, we analysed changes in ventricular CSF volume. Previously, Zahr et al. demonstrated that CSF changes accompany ethanol‐induced disruptions to brain biochemistry measured with MRS in vivo [29, 52, 53]. Ventricular volume changes were observed in this study, but only in females. Importantly, these changes represent additional biological effects rather than methodological confounds, as we corrected for the fraction of the MRS voxel containing CSF in the determination of molecular concentrations.

### 4.2 MRS Changes Are Sex‐Dependent and More Closely Related to Ethanol Intake Than CIE or FSS Exposure

Considerable inter‐individual variability in ethanol intake was observed in this experiment. In addition, a larger amount of drinking escalation occurred during the 6‐week acquisition period, prior to dividing animals into CIE and FSS exposure groups, than expected. At the conclusion of the 6‐week acquisition period, the mean ethanol intake during the 1‐h period was 2.34 ± 0.86 g/kg for males and 2.67 ± 1.11 g/kg for females, which is comparable with the intake levels of 2.3 g/kg in the CIE and FSS exposed male mice at the conclusion of the CIE–FSS period in earlier reports with similar procedures [11]. As a consequence of high variance and limited ability to further increase intake during the second phase of the experiment, the effects of CIE and FSS on ethanol intake were more subtle in this study than in several previous studies [8, 9, 11, 12, 13, 59].

Significant group differences in MRS‐derived concentration changes mirrored patterns observed in ethanol intake and were predominantly between ethanol vapour‐exposed and air‐exposed conditions. Specifically, increases in glutamate, inositol, lactate, taurine and total creatine concentrations were observed to be larger in CIE‐exposed mice compared with air‐exposed controls (Table 3). Two additional group‐related effects were that aspartate concentration change was dependent on both CIE and FSS exposure, and concentration increases in total choline were larger in FSS‐exposed mice.

The substantial variation in ethanol drinking, combined with the primary dependence of MRS and drinking changes on CIE but not FSS exposure, prompted analyses at the individual mouse level, to better understand the potential direct relationship between changes in neurochemical concentrations and ethanol intake. Additionally, one prior study in male rats reported correlations with drinking for glutamine and GABA in the cingulate cortex [60]. Statistically significant positive correlations between changes in intake between Week 1 (the beginning of the acquisition period) and Week 14 (following CIE and FSS exposure) and changes in MRS‐derived concentrations were observed for glutamate, glutamine, taurine and total creatine. As previously established [61, 62], female C57 mice tend to drink more than their male counterparts. Greater inter‐individual variance in drinking was also observed among female mice compared with males in this experiment (Figure 2). Striking qualitative and quantitative differences were observed between males and females in the correlation between MRS changes and escalation of drinking. Neurochemical concentration changes strongly positively correlated with changes in ethanol intake in females. Notably, the observed positive correlations represent moderate effect sizes (*r* ^2^ values of approximately 0.4, Figure 5E–H). In males, correlations between the same chemical species and changes in ethanol drinking were statistically significant but characterized by small to weak effect sizes (*r* ^2^ values in the 0.1–0.3 range), and interestingly, the correlations observed were negative (Figure 5I–L). Combining the sex‐dependent ventricular volume effects with the observed neurochemical changes, findings from this experiment indicate that parenchymal water content may decrease and CSF volume increases in females, but parenchymal water increases in males with no change in CSF volume in males and both sets of changes occur in proportion to ethanol drinking levels. Neurobiological mechanisms underlying the sex differences that we observe are possibly related to enhanced ethanol‐related dopamine response reported in female rodents [63]. Although estrous cycle phase has not been found to substantially correlate with ethanol intake in female mice [16, 17], the effects of sex hormones potentially contribute to sex differences in ethanol intake and nervous system consequences [17]. Moreover, differences in c‐Fos response have been implicated in sex differences in ethanol consumption in C57 mice [64]. Additional research would be beneficial for determining which of these factors, or others, may be contributing to sex differences we find in the dorsal striatum with respect to changes in volitional ethanol drinking.

The correlation analyses shown in Figure 5 demonstrate greater sensitivity in detecting associations between MRS measures and ethanol intake than the group‐level comparisons shown in Figure 4. It is noteworthy that exposure to ethanol alone does not necessarily induce the MRS changes reported here. Indeed, the 16‐h CIE procedures expose mice to greater amounts of ethanol than the 1‐h drinking phases of the experiment, yet the strongest associations observed in this study are between intake during the 1‐h period and MRS‐derived changes. These findings are consistent with MRS changes reflecting risk factors for heavy ethanol intake rather than serving primarily as indicators of ethanol exposure effects. Future studies designed to address this possibility would significantly advance understanding of the neurobiological underpinnings of heavy alcohol use.

### 4.3 Limitations

Certain methodological considerations contribute to the limitations of this study. The MRS voxel covered a large portion of the striatum as well as portions of the lateral ventricles and septal area. This voxel position represents a trade‐off between sensitivity and anatomical specificity. However, the septal area occupied only approximately 9% of the MRS voxel, and we were able to consistently position the voxel dorsal to the nucleus accumbens, providing confidence that our neurochemical measurements reflect changes to the dorsal striatum, with minimal contribution from the ventral striatum. MRS data from comparison brain regions were not acquired, which limits the generalizability of our findings from the dorsal striatum. Somewhat surprisingly, we did not find appreciable effects of our stress treatment (FSS) on either drinking behaviour or neurochemical concentrations. A prior study found FSS to be more effective at increasing ethanol drinking than restraint stress or social defeat stress in a CIE paradigm [65]; however, that experiment only involved male mice, and other work in rodents found that strain and sex differences modified the effects of FSS [66]. A possible explanation for the lack of FSS effects in this study is that mice already achieved an elevated level of drinking prior to FSS introduction, such that FSS was not sufficient to further increase voluntary ethanol consumption. Another limitation was the variable abstinence duration of the mice at the follow‐up MRS timepoint. Prior microdialysis experiments in the striatum of rats indicated that extracellular glutamate returns to control levels by 36 h of ethanol withdrawal, following the conclusion of 6 days of continuously elevated BECs, with approximately 20 g/kg/day of ethanol administered intragastrically [50]. Another microdialysis study of the nucleus accumbens in mice found elevated extracellular glutamate persisting at 7 days after CIE vapour exposure; however, subsequent ethanol gavage did not produce acute effects on extracellular glutamate [51]. Some behavioural studies in rodents have reported peak effects of alcohol abstinence in the timespan from 8 to 24 h [67, 68], whereas others have found behavioural effects of alcohol withdrawal in rats extending past 4 days [69]. Mice in this study received follow‐up MRS sessions between 43.1 and 119.2 h after the most recent ethanol drinking session. We examined the effects of abstinence duration and observed only small non‐significant effects (Figure S2), and permutation testing indicated that abstinence effects did not substantially affect our findings (Figure S3). Although the correlations we observed between changes in neurochemical concentrations and changes in drinking behaviour are promising as noninvasive brain markers of escalated drinking preference, neurobiological interpretations are complicated by the absence of more comprehensive information about the time course of dorsal striatal neurochemical changes following the onset of ethanol abstinence.

## 5 Conclusions

The hypothesis of elevated glutamate/GABA ratios in the dorsal striatum due to CIE was not supported by our data. CIE‐exposed mice exhibited a smaller increase in the concentration of glutamate present in the dorsal striatum than air‐exposed controls. GABA concentrations did not exhibit group effects nor significant correlations with drinking behaviour. Comparisons of our findings with electrophysiology studies [4, 5, 6] are complicated by the fact that MRS detects glutamate and GABA molecules involved in metabolism and protein synthesis, in addition to those involved in neurotransmission. Associations between changes in drinking behaviour and changes in neurochemical concentrations point towards the sensitivity of MRS to characterize drinking escalation trajectories and potentially risk for heavy drinking. Furthermore, in females, correlations were consistent with the hypothesis of increased excitatory tone in the dorsal striatum having an association with heavier ethanol consumption. We found strong differences between female mice and male mice in the direction of these correlations. In part, these sex differences can likely be attributed to underlying factors responsible for sex differences in the escalation of ethanol drinking. The female mice in the study exhibited more variability in their ethanol consumption, suggesting that for female mice, changes in ethanol drinking behaviour are affected by individual factors more than they are in male mice, as indicated by prior CIE studies of female mice [14, 15].

## Author Contributions

**E. B.:** design of mrs experiments, acquisition of study data, formal analysis, writing – original draft. **T. L. C.:** design of mouse procedures, intracranial injections, acquisition of study data, formal analysis. **C. H.:** acquisition of study data. **J. L. T.:** acquisition of study data. **P. N. R.:** design of nmr analysis, acquisition of study data, formal analysis, writing – review and editing. **V. C. C.:** conceptualization, study design, acquisition of study data, formal analysis, writing – review and editing. **C. D. K.:** conceptualization, study design, writing – original draft, writing – review and editing.

## Ethics Statement

Rodent animal procedures were approved by the Institutional Animal Care and Use Committee of Oregon Health and Science University in accordance with the NIH Guide for the Care and Use of Laboratory Animals.

## Conflicts of Interest

The authors declare no conflicts of interest.

## Supporting information


**Figure S1** Weekly 1‐h mean ethanol drinking for male mice (panel A) and female mice (panel B) with lines connecting observation of each individual mouse. Starting at week 8, ethanol drinking occurred every second week (i.e., during FSS weeks). Red lines between week 6 and week 14 indicate mice in the EtOH_vapour_ condition. Panels C–M depict weekly drinking by experimental group and sex. Solid bars indicate mean ethanol consumed; error bars depict standard error of the mean (SEM). Significant effects by 3‐way ANOVA are indicated in the upper left of each panel. Groups were assigned after week 6 and were intentionally balanced by ethanol drinking during the first 6 weeks. It is therefore unsurprising that there are no significant group effects before week 7, however from week 3 to week 5 (E–G) there was a significant main effect of sex, with female mice drinking more ethanol than male mice. During week 8 and week 14 (J and M), EtOH_vapour_ exposure was significantly associated with increased ethanol consumption, consistent with prior studies utilizing CIE‐FSS procedures.

**Figure S2** (A–J) Changes in neurochemical concentrations (mM) are plotted as a function of time (hours) since the final ethanol drinking session (1‐h access to 1 bottle of 15% ethanol). Changes in the (K) Glu/GABA and (L) Glu/Gln ratios are similarly plotted versus time since last ethanol exposure. Each of the MRS‐derived measurements were analysed by linear regression to quantify the effect of abstinence and test for statistical significance. No statistically significant associations were observed (all p‐values, shown in green insets, > 0.05). The largest observed effect of abstinence on change in neurochemical concentration was for total creatine (D) with approximately 7% of variance explained (*R* ^2^ = 0.073).

**Figure S3** For each ∆neurochemical identified in our main findings (Figure 5) as significantly correlated with change in 1‐h/day ethanol drinking (A, Glutamate; B, Glutamine; C, Taurine; D, total Creatine), the consequences of hypothetical effects of abstinence on the association between ∆drinking and ∆neurochemical concentration is simulated. Blue horizontal dashed lines denote the R^2^_drinking_ values of the linear model between ∆neurochemical concentration and ∆drinking for the indicated neurochemical (shown in Figure 5). The green vertical dashed line indicates the observed R^2^_abstinence_ of the linear model between ∆neurochemical concentration and abstinence length (hours). For each neurochemical, 1000 pseudorandom permutations of mouse index, over the set of actual abstinence durations, were generated. Each permuted dataset was adjusted to introduce a synthetic R^2^_abstinence_ of 0.01, 0.05, 0.10, 0.15, or 0.20 by adding the term [(synthetic R^2^ × observed variance at follow up)^1/2^ × ((permuted abstinence − mean{permuted abstinence}) /SD{permuted abstinence})] to the follow up neurochemical concentrations as a function of permuted abstinence. Black circles and error bars indicate the mean and ±1 standard deviation range of the 1000 R^2^_drinking_ values resulting from permutation of abstinence duration, for each simulated abstinence effect size. This approach preserves the true multi‐modal distribution of abstinence durations in our sample. For each of the four graphs, the trend is towards negatively biased apparent R^2^_drinking_, as R^2^_abstinence_ increases, indicating that the observed correlations between ∆neurochemical concentration and ∆drinking are more likely to be underestimated, rather than inflated, as a result of variance introduced by abstinence duration.

**Figure S4** Neurochemical concentrations at follow up for 5 neurochemicals and the ratio of Glu to GABA. Glutamate (A), GABA (B), and their ratio (C) did not exhibit effects of ethanol vapour or stress conditions, nor a significant sex effect. The strongest effects of group on concentrations at follow up were observed for glutamine (D) with males showing higher glutamine concentrations (main effect of sex, *p* = 0.01). Aspartate (E) showed a CIE‐by‐FSS interaction (*p* = 0.02), with males in the EtOH_vapour_ + stress condition having the highest aspartate concentration. Lactate (F) was lower in EtOH_vapour_ mice compared to air‐exposed controls (main effect of CIE, *p* = 0.04). * *p* < 0.05, ** *p* < 0.01.

**Figure S5** Comparison between five MRS concentration datasets from the mouse striatum. 1—The dorsal striatal MRS neurochemical concentration estimates for the present study at baseline and 2—at follow up in grey circles, connected by grey lines (red horizonal bars indicate means, black horizontal bars indicate ± 1 standard deviations). 3—Dorsal striatal MRS concentrations from a separate cohort of ethanol‐naïve genetically diverse DO mice [1] (orange circles) acquired at our site with similar acquisition parameters as were used in the present study. 4—Striatal concentration estimates from a prior study by Chassain et al. [2] in C57BL/6 mice with means (dark red squares) and standard deviations (error bars) for the 9 reported neurochemicals in control mice. 5—Striatal concentration estimates from an additional prior study by Tkáč et al. [3] also using striatal MRS in C57BL/6 mice. Baseline neurochemical concentrations for the mice utilized in this study are consistent with striatal neurochemical concentrations determined in other studies of ethanol‐naïve mice.

**Table S1** Results from ANOVA of group effects on metabolite concentrations at follow up.

**Data S1** Supporting Information.

**Data S2** Supporting Information.

**Data S3** Supporting Information.

## Data Availability

The data that supports the findings of this study are available in the supporting Information of this article.
