# Supplementary material for: Escalation of Ethanol Drinking in Mice Is Associated With Neurochemical Changes in the Dorsal Striatum
Source: Addict Biol. 2025 Nov 25;30(12):e70101. doi: 10.1111/adb.70101 (PMC12646714; doi:10.1111/adb.70101)
Supplement: Supplementary file 6 — Table S1 Results from ANOVA of group effects on metabolite concentrations at follow up. [file ADB-30-e70101-s004.docx]

Table S1: Results from ANOVA of group effects on metabolite concentrations at follow up

|  | CIE | | FSS | | sex | | CIE by FSS | | CIE by sex | | FSS by sex | | CIE by FSS by sex | |
| --- | --- | --- | --- | --- | --- | --- | --- | --- | --- | --- | --- | --- | --- | --- |
| Neurochemical | **F-statistic** | **p -value** | **F-statistic** | **p -value** | **F-statistic** | **p -value** | **F-statistic** | **p -value** | **F-statistic** | **p -value** | **F-statistic** | **p -value** | **F-statistic** | **p -value** |
| Alanine | 0.24 | 0.63 | 2.54 | 0.12 | 0.77 | 0.39 | 0.45 | 0.51 | 0.00 | 0.99 | 2.71 | 0.11 | 0.30 | 0.59 |
| Aspartate | 1.10 | 0.30 | 0.35 | 0.56 | 0.28 | 0.60 | 6.01 | **0.02** | 3.49 | 0.07 | 0.75 | 0.39 | 0.57 | 0.46 |
| GABA | 0.45 | 0.51 | 0.03 | 0.86 | 0.16 | 0.69 | 0.53 | 0.47 | 0.63 | 0.43 | 0.00 | 0.99 | 0.07 | 0.79 |
| Glutamine | 2.37 | 0.13 | 0.00 | 0.94 | 7.27 | **0.01** | 0.16 | 0.69 | 0.15 | 0.69 | 1.65 | 0.21 | 0.17 | 0.68 |
| Glutamate | 0.31 | 0.57 | 0.15 | 0.70 | 0.09 | 0.76 | 1.44 | 0.24 | 1.48 | 0.23 | 0.02 | 0.90 | 0.00 | 0.96 |
| Inositol | 1.14 | 0.29 | 0.04 | 0.83 | 0.26 | 0.61 | 0.09 | 0.77 | 0.21 | 0.65 | 0.35 | 0.56 | 0.00 | 0.99 |
| Lactate | 4.76 | **0.04** | 0.55 | 0.46 | 0.63 | 0.43 | 1.68 | 0.20 | 0.14 | 0.71 | 0.02 | 0.88 | 0.08 | 0.79 |
| Taurine | 0.33 | 0.57 | 0.00 | 0.99 | 0.53 | 0.47 | 0.38 | 0.54 | 0.20 | 0.66 | 0.01 | 0.94 | 0.02 | 0.89 |
| total Choline | 0.02 | 0.90 | 0.03 | 0.86 | 0.00 | 0.95 | 0.83 | 0.37 | 0.20 | 0.66 | 0.08 | 0.78 | 0.13 | 0.72 |
| NAA | 0.00 | 0.96 | 0.61 | 0.44 | 0.48 | 0.49 | 0.72 | 0.40 | 0.67 | 0.42 | 0.08 | 0.78 | 0.02 | 0.89 |
| total Creatine | 0.47 | 0.49 | 0.02 | 0.89 | 0.53 | 0.47 | 0.18 | 0.68 | 0.32 | 0.57 | 0.06 | 0.80 | 0.01 | 0.91 |
| Glu / GABA | 2.10 | 0.15 | 0.07 | 0.79 | 0.26 | 0.61 | 0.00 | 0.97 | 0.00 | 0.98 | 0.01 | 0.93 | 0.04 | 0.84 |
| Glu / Gln | 4.05 | 0.05 | 0.11 | 0.74 | 7.19 | **0.01** | 0.65 | 0.42 | 0.21 | 0.65 | 0.91 | 0.35 | 0.18 | 0.67 |

Bold and underlined numbers indicate p-values that are significant at α=0.05.
