# Supplementary material for: Escalation of Ethanol Drinking in Mice Is Associated With Neurochemical Changes in the Dorsal Striatum
Source: Addict Biol. 2025 Nov 25;30(12):e70101. doi: 10.1111/adb.70101 (PMC12646714; doi:10.1111/adb.70101)
Supplement: Supplementary file 7 — Data S1 Supporting Information. [file ADB-30-e70101-s008.docx]

***Escalation of ethanol drinking in mice is associated with neurochemical changes in the dorsal striatum***

Eric Baetscher, Timothy L. Carlson, Connor Hilts, Jade L. Thomas, Patrick N. Reardon, Vergina C. Cuzon Carlson, Christopher D. Kroenke

**Supplementary information**

**
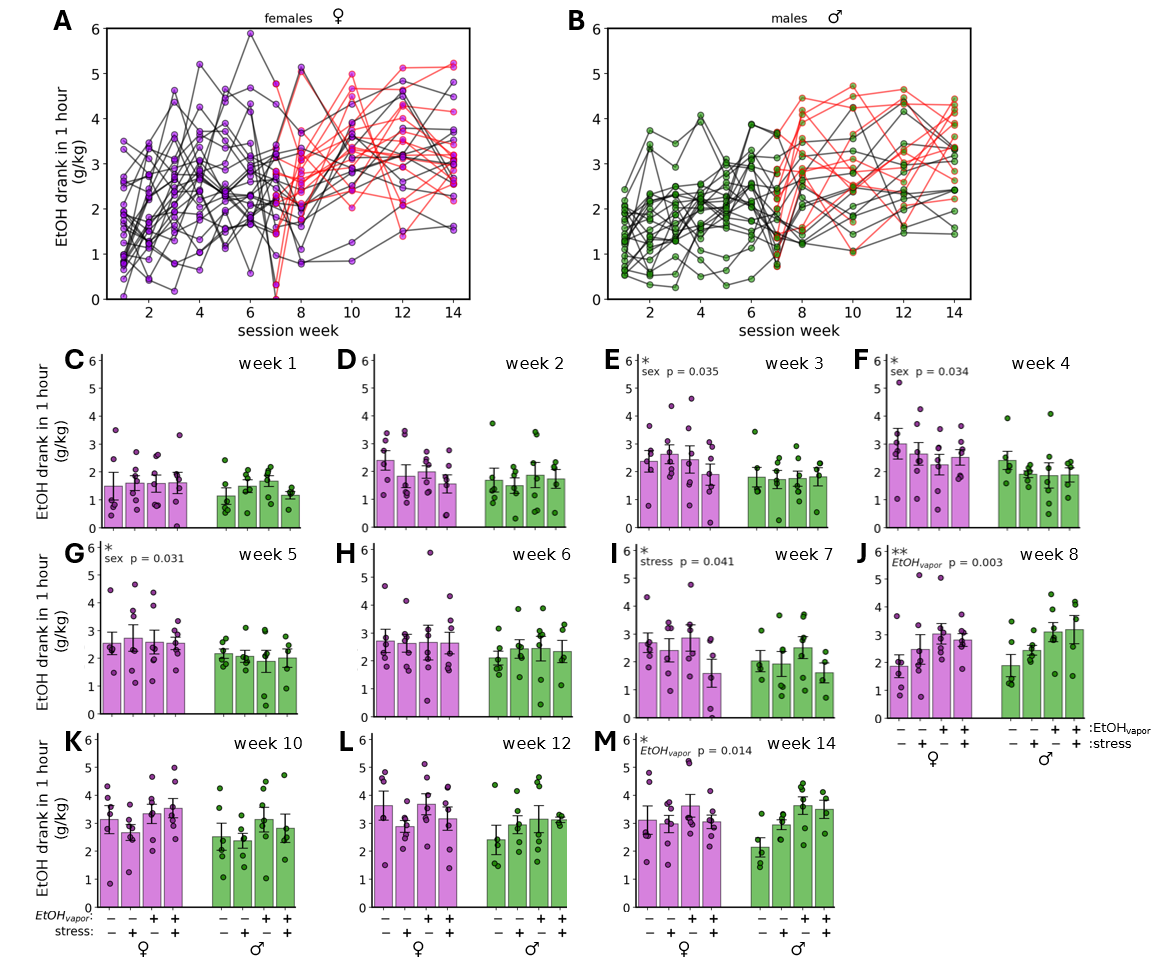
**

Figure S1: Weekly 1-hour mean ethanol drinking for male mice (panel A) and female mice (panel B) with lines connecting observation of each individual mouse. Starting at week 8, ethanol drinking occurred every second week (that is, during FSS weeks). Red lines between week 6 and week 14 indicate mice in the EtOH_vapor_ condition. Panels C – M depict weekly drinking by experimental group and sex. Solid bars indicate mean ethanol consumed; error bars depict standard error of the mean (SEM). Significant effects by 3-way ANOVA are indicated in the upper left of each panel. Groups were assigned after week 6 and were intentionally balanced by ethanol drinking during the first 6 weeks. It is therefore unsurprising that there are no significant group effects before week 7, however from week 3 to week 5 (E – G) there was a significant main effect of sex, with female mice drinking more ethanol than male mice. During week 8 and week 14 (J and M), EtOH_vapor_ exposure was significantly associated with increased ethanol consumption, consistent with prior studies utilizing CIE-FSS procedures.

**
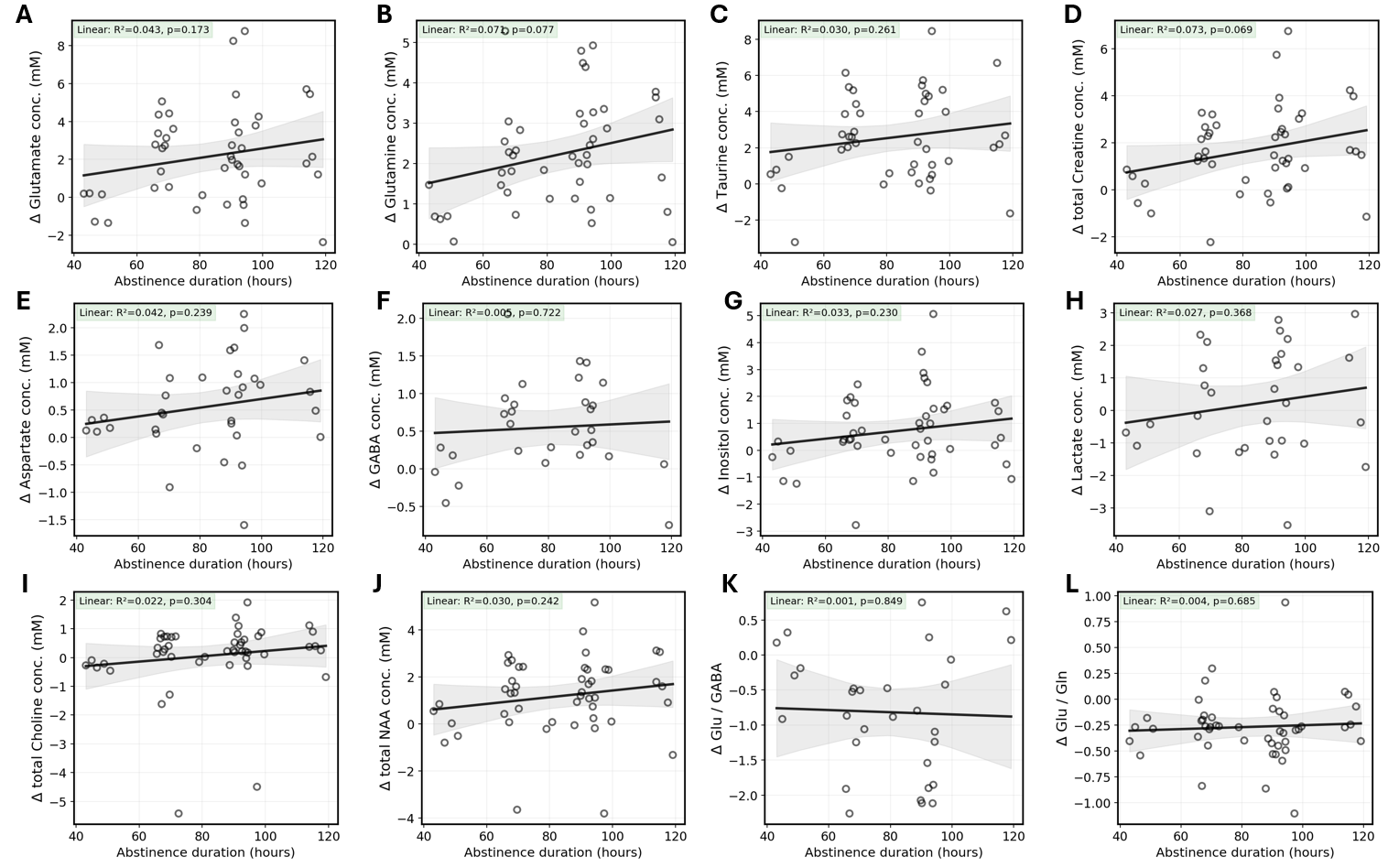
**

Figure S2: (A-J) Changes in neurochemical concentrations (mM) are plotted as a function of time (hours) since the final ethanol drinking session (1-hour access to 1 bottle of 15% ethanol). Changes in the (K) Glu/GABA and (L) Glu/Gln ratios are similarly plotted versus time since last ethanol exposure. Each of the MRS-derived measurements were analyzed by linear regression to quantify the effect of abstinence and test for statistical significance. No statistically significant associations were observed (all p-values, shown in green insets, >0.05). The largest observed effect of abstinence on change in neurochemical concentration was for total creatine (D) with approximately 7% of variance explained (R^2^=0.073).


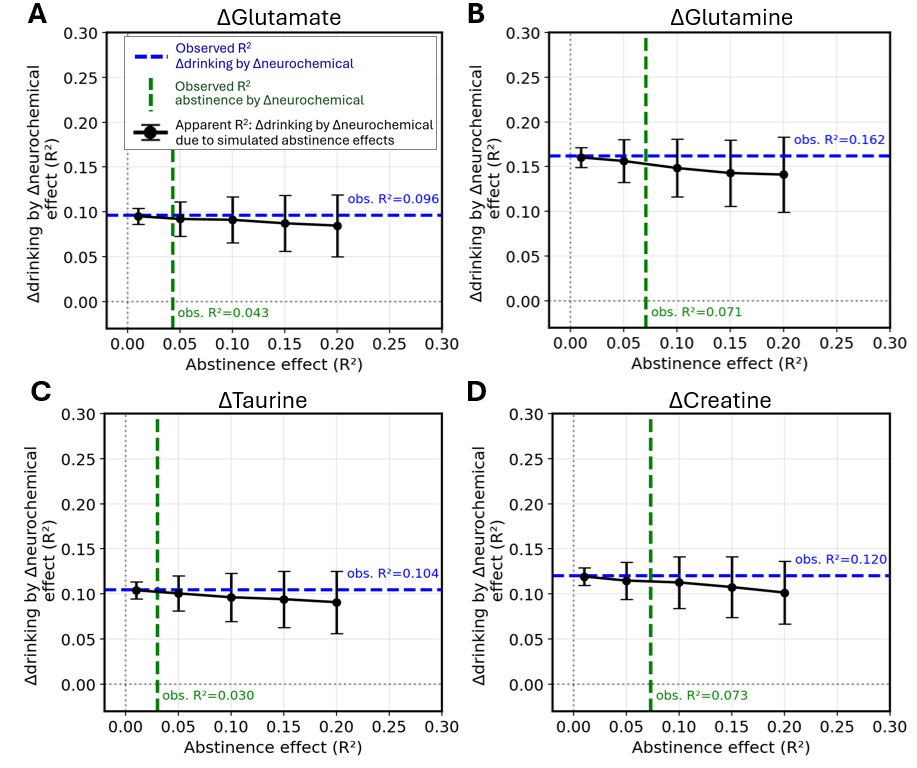


Figure S3: For each ∆neurochemical identified in our main findings (Figure 5) as significantly correlated with change in 1-hour/day ethanol drinking (A, Glutamate; B, Glutamine; C, Taurine; D, total Creatine), the consequences of hypothetical effects of abstinence on the association between ∆drinking and ∆neurochemical concentration is simulated. Blue horizontal dashed lines denote the R^2^_drinking_ values of the linear model between ∆neurochemical concentration and ∆drinking for the indicated neurochemical (shown in Figure 5). The green vertical dashed line indicates the observed R^2^_abstinence_ of the linear model between ∆neurochemical concentration and abstinence length (hours). For each neurochemical, 1000 pseudorandom permutations of mouse index, over the set of actual abstinence durations, were generated. Each permuted dataset was adjusted to introduce a synthetic R^2^_abstinence_ of 0.01, 0.05, 0.10, 0.15, or 0.20 by adding the term [(synthetic R^2^ × observed variance at follow up)^1/2^ × ((permuted abstinence – mean{permuted abstinence}) / SD{permuted abstinence})] to the follow up neurochemical concentrations as a function of permuted abstinence. Black circles and error bars indicate the mean and ±1 standard deviation range of the 1000 R^2^_drinking_ values resulting from permutation of abstinence duration, for each simulated abstinence effect size. This approach preserves the true multi-modal distribution of abstinence durations in our sample. For each of the four graphs, the trend is towards negatively biased apparent R^2^_drinking_, as R^2^_abstinence_ increases, indicating that the observed correlations between ∆neurochemical concentration and ∆drinking are more likely to be underestimated, rather than inflated, as a result of variance introduced by abstinence duration.

Table S1: Results from ANOVA of group effects on metabolite concentrations at follow up

|  | CIE | | FSS | | sex | | CIE by FSS | | CIE by sex | | FSS by sex | | CIE by FSS by sex | |
| --- | --- | --- | --- | --- | --- | --- | --- | --- | --- | --- | --- | --- | --- | --- |
| Neurochemical | **F-statistic** | **p -value** | **F-statistic** | **p -value** | **F-statistic** | **p -value** | **F-statistic** | **p -value** | **F-statistic** | **p -value** | **F-statistic** | **p -value** | **F-statistic** | **p -value** |
| Alanine | 0.24 | 0.63 | 2.54 | 0.12 | 0.77 | 0.39 | 0.45 | 0.51 | 0.00 | 0.99 | 2.71 | 0.11 | 0.30 | 0.59 |
| Aspartate | 1.10 | 0.30 | 0.35 | 0.56 | 0.28 | 0.60 | 6.01 | **0.02** | 3.49 | 0.07 | 0.75 | 0.39 | 0.57 | 0.46 |
| GABA | 0.45 | 0.51 | 0.03 | 0.86 | 0.16 | 0.69 | 0.53 | 0.47 | 0.63 | 0.43 | 0.00 | 0.99 | 0.07 | 0.79 |
| Glutamine | 2.37 | 0.13 | 0.00 | 0.94 | 7.27 | **0.01** | 0.16 | 0.69 | 0.15 | 0.69 | 1.65 | 0.21 | 0.17 | 0.68 |
| Glutamate | 0.31 | 0.57 | 0.15 | 0.70 | 0.09 | 0.76 | 1.44 | 0.24 | 1.48 | 0.23 | 0.02 | 0.90 | 0.00 | 0.96 |
| Inositol | 1.14 | 0.29 | 0.04 | 0.83 | 0.26 | 0.61 | 0.09 | 0.77 | 0.21 | 0.65 | 0.35 | 0.56 | 0.00 | 0.99 |
| Lactate | 4.76 | **0.04** | 0.55 | 0.46 | 0.63 | 0.43 | 1.68 | 0.20 | 0.14 | 0.71 | 0.02 | 0.88 | 0.08 | 0.79 |
| Taurine | 0.33 | 0.57 | 0.00 | 0.99 | 0.53 | 0.47 | 0.38 | 0.54 | 0.20 | 0.66 | 0.01 | 0.94 | 0.02 | 0.89 |
| total Choline | 0.02 | 0.90 | 0.03 | 0.86 | 0.00 | 0.95 | 0.83 | 0.37 | 0.20 | 0.66 | 0.08 | 0.78 | 0.13 | 0.72 |
| NAA | 0.00 | 0.96 | 0.61 | 0.44 | 0.48 | 0.49 | 0.72 | 0.40 | 0.67 | 0.42 | 0.08 | 0.78 | 0.02 | 0.89 |
| total Creatine | 0.47 | 0.49 | 0.02 | 0.89 | 0.53 | 0.47 | 0.18 | 0.68 | 0.32 | 0.57 | 0.06 | 0.80 | 0.01 | 0.91 |
| Glu / GABA | 2.10 | 0.15 | 0.07 | 0.79 | 0.26 | 0.61 | 0.00 | 0.97 | 0.00 | 0.98 | 0.01 | 0.93 | 0.04 | 0.84 |
| Glu / Gln | 4.05 | 0.05 | 0.11 | 0.74 | 7.19 | **0.01** | 0.65 | 0.42 | 0.21 | 0.65 | 0.91 | 0.35 | 0.18 | 0.67 |

Bold and underlined numbers indicate p-values that are significant at α=0.05.


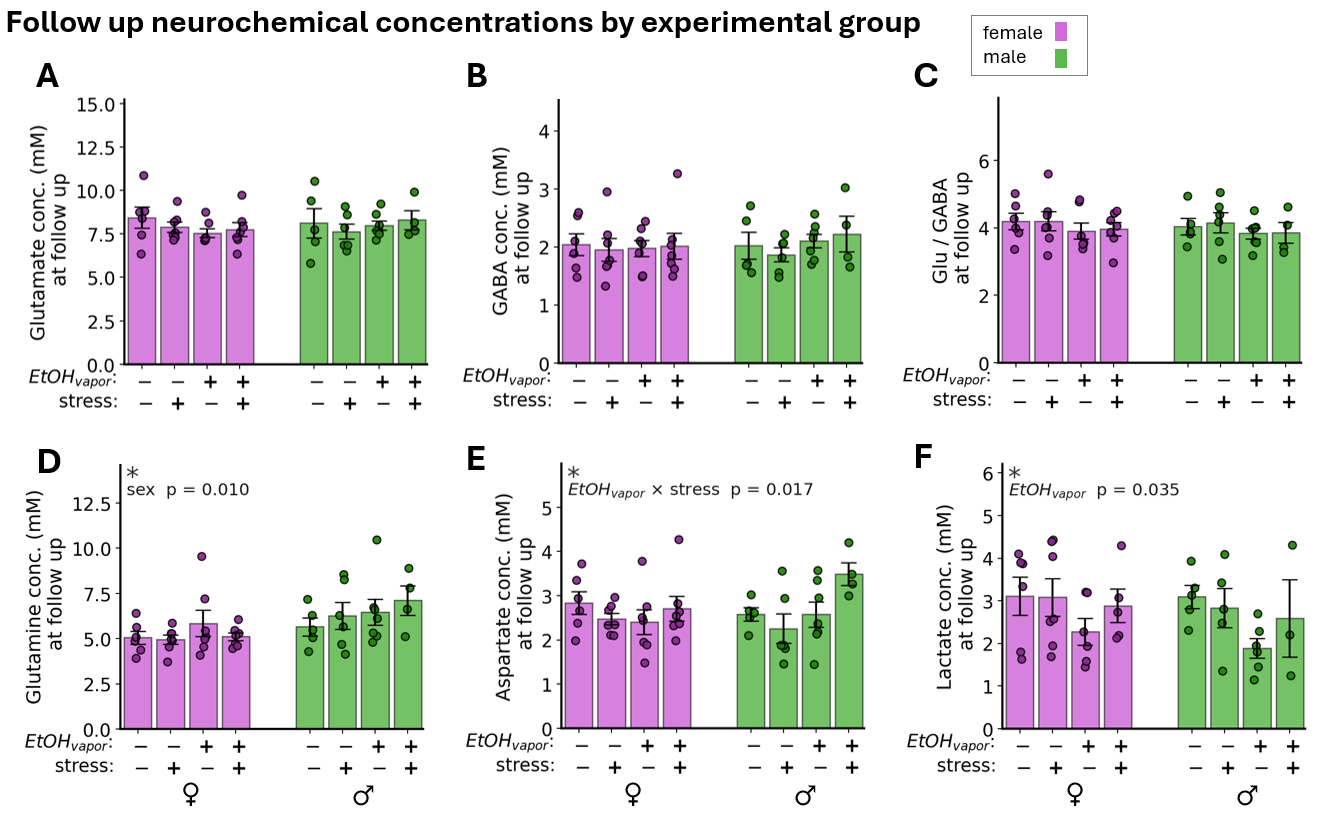


Figure S3: Neurochemical concentrations at follow up for 5 neurochemicals and the ratio of Glu to GABA. Glutamate (A), GABA (B), and their ratio (C) did not exhibit effects of ethanol vapor or stress conditions, nor a significant sex effect. The strongest effects of group on concentrations at follow up were observed for glutamine (D) with males showing higher glutamine concentrations (main effect of sex, p = 0.01). Aspartate (E) showed a CIE-by-FSS interaction (p = 0.02), with males in the EtOH_vapor_ + stress condition having the highest aspartate concentration. Lactate (F) was lower in EtOH_vapor_ mice compared to air-exposed controls (main effect of CIE, p = 0.04). * p < 0.05, ** p < 0.01


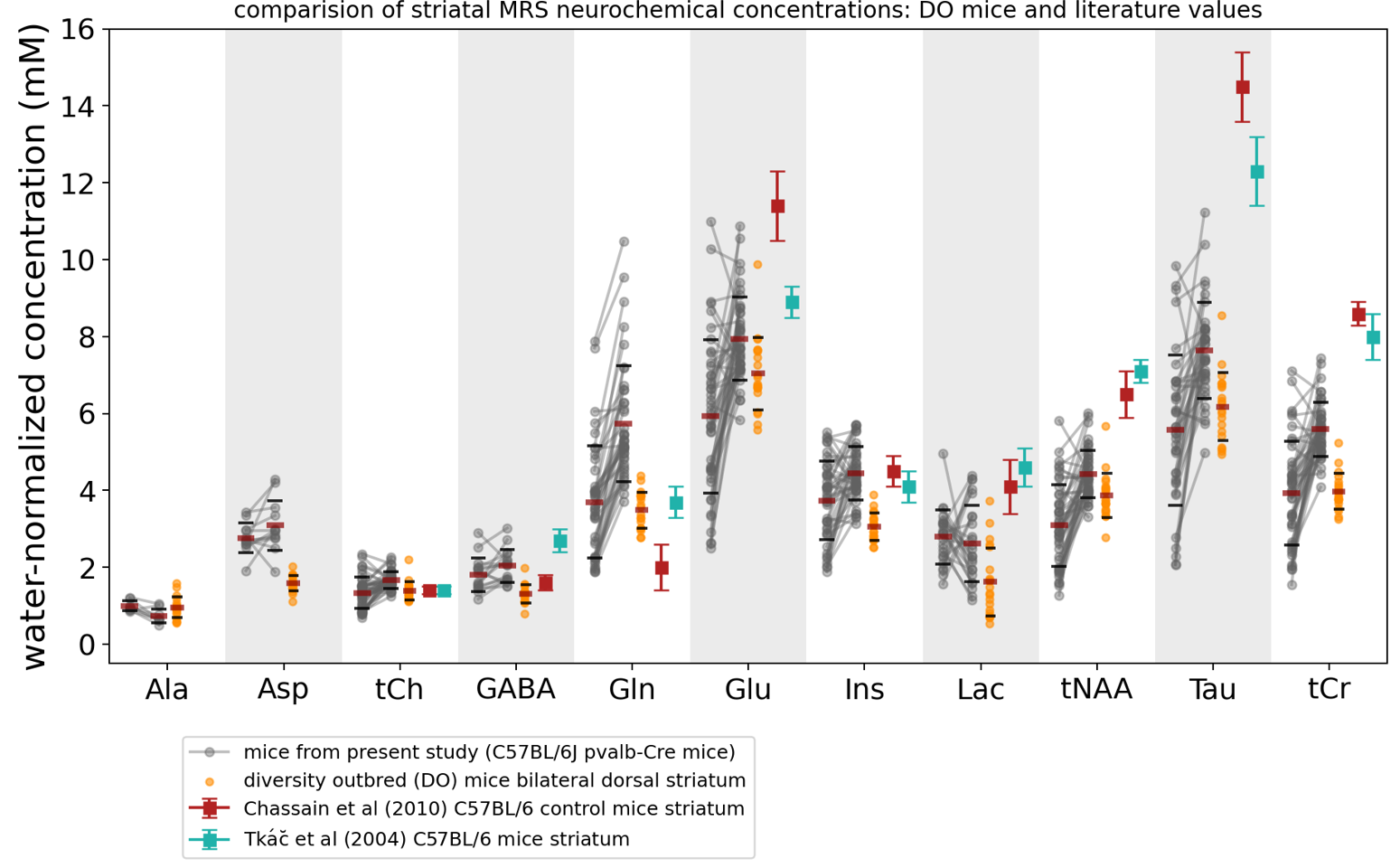


Figure S4: Comparison between five MRS concentration datasets from the mouse striatum. 1 – The dorsal striatal MRS neurochemical concentration estimates for the present study at baseline and 2 – at follow up in gray circles, connected by gray lines (red horizonal bars indicate means, black horizontal bars indicate ± 1 standard deviations). 3 – Dorsal striatal MRS concentrations from a separate cohort of ethanol-naïve genetically diverse DO mice [1] (orange circles) acquired at our site with similar acquisition parameters as were used in the present study. 4 – Striatal concentration estimates from a prior study by Chassain et al. [2] in C57BL/6 mice with means (dark red squares) and standard deviations (error bars) for the 9 reported neurochemicals in control mice. 5 – Striatal concentration estimates from an additional prior study by Tkáč et al. [3] also using striatal MRS in C57BL/6 mice. Baseline neurochemical concentrations for the mice utilized in this study are consistent with striatal neurochemical concentrations determined in other studies of ethanol-naïve mice.

**References**

1. Saul, M.C., et al., *High-diversity mouse populations for complex traits.* Trends in Genetics, 2019. **35**(7): p. 501-514.

2. Chassain, C., et al., *Metabolic changes detected in vivo by 1H MRS in the MPTP‐intoxicated mouse.* NMR in Biomedicine, 2010. **23**(6): p. 547-553.

3. Tkáč, I., et al., *Regional sex differences in neurochemical profiles of healthy mice measured by magnetic resonance spectroscopy at 9.4 tesla.* Frontiers in Neuroscience, 2023. **17**: p. 1278828.
